# Quantitative Transcriptomics Reveals the Growth- and Nutrient-Dependent Response of a Streamlined Marine Methylotroph to Methanol and Naturally Occurring Dissolved Organic Matter

**DOI:** 10.1128/mBio.01279-16

**Published:** 2016-11-22

**Authors:** Scott M. Gifford, Jamie W. Becker, Oscar A. Sosa, Daniel J. Repeta, Edward F. DeLong

**Affiliations:** a Department of Marine Sciences, University of North Carolina at Chapel Hill, Chapel Hill, North Carolina, USA; b Department of Civil and Environmental Engineering, Massachusetts Institute of Technology, Cambridge, Massachusetts, USA; c Daniel K. Inouye Center for Microbial Oceanography: Research and Education (C-MORE), University of Hawaii, Honolulu, Hawaii, USA; d Department of Marine Chemistry and Geochemistry, Woods Hole Oceanographic Institution, Woods Hole, Massachusetts, USA

## Abstract

The members of the OM43 clade of *Betaproteobacteria* are abundant coastal methylotrophs with a range of carbon-utilizing capabilities. However, their underlying transcriptional and metabolic responses to shifting conditions or different carbon substrates remain poorly understood. We examined the transcriptional dynamics of OM43 isolate NB0046 subjected to various inorganic nutrient, vitamin, and carbon substrate regimes over different growth phases to (i) develop a quantitative model of its mRNA content; (ii) identify transcriptional markers of physiological activity, nutritional state, and carbon and energy utilization; and (iii) identify pathways involved in methanol or naturally occurring dissolved organic matter (DOM) metabolism. Quantitative transcriptomics, achieved through addition of internal RNA standards, allowed for analyses on a transcripts-per-cell scale. This streamlined bacterium exhibited substantial shifts in total mRNA content (ranging from 1,800 to 17 transcripts cell^−1^ in the exponential and deep stationary phases, respectively) and gene-specific transcript abundances (>1,000-fold increases in some cases), depending on the growth phase and nutrient conditions. Carbon metabolism genes exhibited substantial dynamics, including those for ribulose monophosphate, tricarboxylic acid (TCA), and proteorhodopsin, as well as methanol dehydrogenase (*xoxF*), which, while always the most abundant transcript, increased from 5 to 120 transcripts cell^−1^ when cultures were nutrient and vitamin amended. In the DOM treatment, upregulation of TCA cycle, methylcitrate cycle, vitamin, and organic phosphorus genes suggested a metabolic route for this complex mixture of carbon substrates. The genome-wide inventory of transcript abundances produced here provides insight into a streamlined marine bacterium’s regulation of carbon metabolism and energy flow, providing benchmarks for evaluating the activity of OM43 populations *in situ*.

## IMPORTANCE

Bacteria exert a substantial influence on marine organic matter flux, yet the carbon components targeted by specific bacterial groups, as well as how those groups’ metabolic activities change under different conditions, are not well understood. Gene expression studies of model organisms can identify these responses under defined conditions, which can then be compared to environmental transcriptomes to elucidate *in situ* activities. This integration, however, is limited by the data’s relative nature. Here, we report the fully quantitative transcriptome of a marine bacterium, providing a genome-wide survey of cellular transcript abundances and how they change with different states of growth, nutrient conditions, and carbon substrates. The results revealed the dynamic metabolic strategies this methylotroph has for processing both simple one-carbon compounds and the complex multicarbon substrates of naturally derived marine organic matter and provide baseline quantitative data for identifying their *in situ* activities and impact on the marine carbon cycle.

## INTRODUCTION

Microbial activities are major drivers of nutrient cycles and energy transfer in marine ecosystems, and the ability to monitor these activities *in situ* has been enhanced by recently developed genome-enabled technologies. An important application for these analyses is understanding how the numerous genes for processing carbon compounds in bacterial genomes are regulated in response to cell physiology and environmental conditions to ultimately influence carbon flux through marine environments. Focusing these omic analyses on model organisms that represent groups with specific carbon-processing capabilities should enable us to better predict how the diverse carbon pool in marine environments is metabolized by bacterioplankton communities and how those roles may change under different conditions.

One functional group of bacteria increasingly recognized as having an impact on the marine carbon cycle is the methylotrophs, microorganisms capable of metabolizing single-carbon substrates (1, 2). The presence of methylotrophs in the marine environment has been recognized for several decades owing to their enrichment and cultivation from seawater samples (3) and more recently in metagenomic surveys (4). Recent investigations have further expanded the potential role of methylotrophs in the marine environment to include high-molecular-weight (HMW) dissolved organic matter (DOM) cycling (5) and phytoplankton-bacteria interactions (6). The sources of methanol supporting these populations in the marine environment are not well understood, but a recent study by Mincer and Aicher (7) showed that several marine phytoplankton taxa can release micromolar concentrations of methanol. Additionally, analyses of methanol standing stocks in seawater has revealed nanomolar concentrations with short turnover times, suggesting that methylotrophs may have a substantial role in the flux of marine carbon (8–10).

In coastal environments, members of the OM43 clade of *Betaproteobacteria* of the family *Methylophilaceae* have emerged as important marine methylotrophs. First identified by Rappé et al. (11), these organisms are a common component of coastal bacterial communities (12) and can substantially increase in relative abundance during phytoplankton blooms (13). The first OM43 genome sequences indicated the presence of highly streamlined genomes in this group (1.3 Mbp encoding 1,377 genes) and suggested that members of the clade might be obligate methylotrophs because of an incomplete tricarboxylic acid (TCA) cycle (14, 15). Halsey et al. (16) demonstrated that OM43 strains are not solely limited to methanol as a substrate, as they can metabolize a range of C1 compounds, including dimethylsulfoniopropionate and trimethylamine oxide. Furthermore, they found that the availability of these substrates shifted the metabolic fate of methanol toward either dissimilatory or assimilatory processes. OM43 isolates have recently been obtained from ultraoligotrophic environments (17), and a closely related sister clade has been isolated from freshwater environments (18), with both isolates also having streamlined genomes and a metabolic range similar to that of previously described OM43 strains. Evidence of a further expanded metabolic range in OM43 clade methylotrophs was recently demonstrated for multiple OM43 strains that were isolated via growth on naturally derived HMW DOM (19). Growth assays of these isolates showed that they could reach substantial cell densities (>10^6^ ml^−1^) on either methanol or HMW DOM. However, the genes enabling this expanded metabolic range, as well as their regulation under different environmental conditions, remain unknown.

Complementing these laboratory observations, environmental surveys are revealing that members of the OM43 clade are active, dynamic members of coastal communities. Metatranscriptomes and metaproteomes revealed that OM43 *xoxF-*type methanol dehydrogenases are some of the most highly transcribed genes and among the most abundant proteins in coastal systems (20, 21). Furthermore, *in situ* observations suggest that these genes are dynamically regulated, exhibiting substantial shifts in their transcript profiles over both seasonal and diel time scales (22). For example, OM43 *xoxF*-type methanol dehydrogenase transcripts had higher seasonal abundances in the fall and winter but minimal day-night differences, while in contrast, transcripts for ribosomal proteins and elongation factors displayed some of the largest day-night differences observed in the entire microbial community (22). These results suggest that OM43 members may tightly tune their transcriptome to the environmental conditions of coastal ecosystems, including temporal shifts in nutrient availability and primary production. However, it is not known which environmental factors are responsible for driving OM43 transcription dynamics.

There is thus a need for experimentally validated, quantitative transcriptional markers that can be used as reporters of the physiological and metabolic state of OM43 clade cells under different conditions, particularly those indicating growth phase, nutrient stress, and carbon substrate utilization. *In situ* quantitative omic techniques have recently been developed that allow for the calculation of absolute gene, transcript, or protein abundances in marine samples on a per-environmental-unit basis (23–26). However, as these environmental gene inventories increase, interpreting their biological or ecological meaning requires a knowledge of how cellular transcript abundances map to the activity, metabolism, and physiology of cell populations. Comparative quantitative transcriptomics of model organisms grown under defined conditions can identify transcriptional markers of physiological or metabolic activity and provide an inventory of cellular transcript abundances to aid in the interpretation of microbial activities *in situ* via metatranscriptomic surveys.

In this study, we applied quantitative transcriptomics to an analysis of OM43 clade strain NB0046 to (i) understand the quantitative nature of a transcriptome from a streamlined marine bacterium, including total mRNA content and genome-wide cellular transcript abundances, and how they change with growth phase; (ii) identify transcriptional benchmarks of physiological activity, metabolism, and the nutritional state; and (iii) explore the metabolic pathways and regulation involved in the metabolism of methanol and naturally occurring HMW DOM.

## RESULTS AND DISCUSSION

### Growth response to nutrient amendments.

OM43 strain NB0046 was originally isolated off the coast of Massachusetts in the United States via dilution to extinction in a seawater medium enriched with naturally occurring HMW DOM (19). For the experiments described in this report, NB0046 was grown in a basal medium consisting of Sargasso seawater sterilized by tangential-flow filtration (TFF) through a 1-kDa membrane and amended as follows. For regime I, the inorganic-nutrient-deplete condition, no inorganic nutrients or vitamins were added. For regime II, the inorganic-nutrient-amended condition, 400 µM NH_4_^+^ and 30 µM PO_4_^3−^ were added. Regime III consisted of inorganic-nutrient- and vitamin-amended conditions with 400 µM NH_4_^+^, 30 µM PO_4_^3−^, and a vitamin mixture with shaking. Regime IV consisted of inorganic-nutrient- and vitamin-amended conditions with 400 µM NH_4_^+^, 30 µM PO_4_^3−^, and a vitamin mixture without shaking. For a carbon source, the cultures were amended with either 50 µM methanol or various concentrations of HMW DOM (see Materials and Methods).

**FIG 1:**
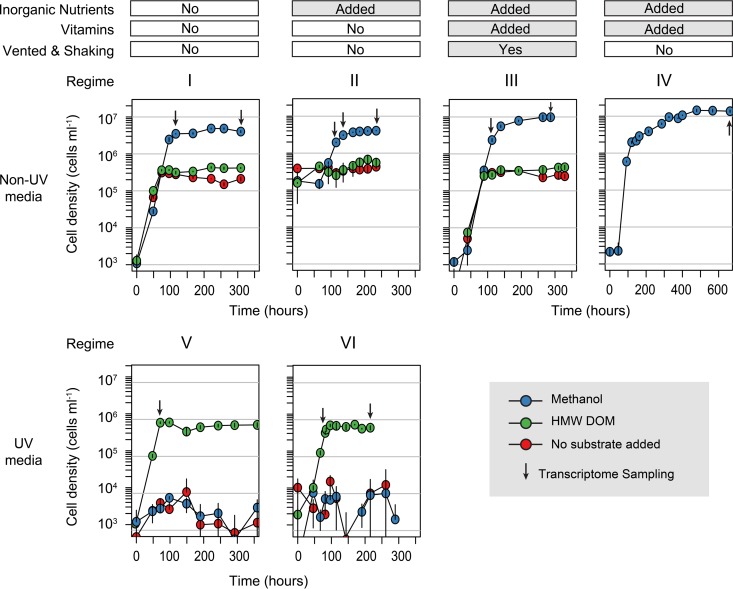
Growth of OM43 clade betaproteobacterial strain NB0046 in Sargasso seawater medium amended with different nutrient and carbon substrates. Error bars represent the SD of triplicate samples for each treatment. For regime IV, the total length of the experiment was twice that of the others. Inorganic nutrients, 30 µM phosphate and 400 µM ammonium; vitamins, AMS1 vitamin mixture; vented and shaking, incubation bottles were shaken at 60 rpm and their caps loosed to increase ventilation of the medium. Arrows indicate sampling points from which transcriptomes were generated.

Cultures amended with methanol reached a cell density (3 × 10^6^ ml^−1^; Fig. 1) typical of growth in Sargasso seawater medium, as previously described (19), which was lower than that observed for other OM43 clade cultures grown in coastal seawater medium (16). The lower cell density appears not to be the result of reduced inorganic nutrient concentrations, since the addition of NH_4_^+^ and PO_4_^3−^ did not significantly increase the cell yield (Fig. 1). The addition of an AMS1 vitamin mixture (27), however, did increase the concentrations to >10^7^ cells ml^−1^. Shaking the nutrient- and vitamin-enriched cultures at 60 rpm did not further increase the cell yield (Fig. 1). The growth rates of the methanol-amended cultures in exponential phase ranged from 0.06 to 0.09 h^−1^, similar to previous observations of OM43 isolates (14, 16).

NB0046 growth on HMW DOM varied significantly, depending on the experimental conditions. Robust growth of this strain was consistently observed in high concentrations of HMW DOM when it was grown in small-volume chambers (200-µl to 1-ml wells). However, when the batch culture volume was increased (from 8 to 250 ml), growth on HMW DOM was severely diminished (see Fig. S1 in the supplemental material) and often did not significantly differ from that of the non-carbon-amended control, although the HMW DOM treatments exhibited significantly greater cell yields than the non-carbon-amended cultures later into stationary phase under regimes I and III (*t* test, *P* < 0.05; Fig. 1). We considered the possibility that ventilation may be one reason for the observed difference between the multiwell plates and the bottles; however, when the cells were shaken in 500-ml bottles with vented lids in regime III, there was no substantial increase in cell yields over regimes I and II for the HMW DOM amendments. Given the low growth yields on HMW DOM in large-volume cultures of regimes I, II, and III, we were unable to capture these bacteria for transcriptome analysis. However, when the seawater medium was UV oxidized (prior to nutrient or substrate additions) to reduce the background concentration of endogenous DOM, NB0046 exhibited significant growth only when the medium was amended with HMW DOM and did not grow in the non-carbon-amended control or methanol treatment (Fig. 1). In order to gain more insight into OM43 HMW DOM metabolism, we therefore grew and collected cells for transcriptional analysis in UV-oxidized seawater medium amended as follows. Regime V consisted of UV-oxidized seawater with no inorganic nutrient or vitamin amendment, and regime VI consisted of UV-oxidized seawater amended with inorganic nutrients (400 µM NH_4_^+^ and 30 µM PO_4_^3−^).

The treatments again included methanol, HMW DOM, or a non-carbon-amended control. In these experiments, inorganic nutrients once again did not substantially increase maximum cell yields and there was no detectable growth in the non-carbon-amended control or methanol additions (Fig. 1).

### Direct stimulation of growth by methanol and HMW DOM amendments.

Our cell yields under non-carbon-amended conditions were consistent with previous reports that OM43 clade members can reach substantial cell densities (≥10^5^ ml^−1^) when grown in a naturally derived seawater medium that is not carbon amended (14, 19). Recent *in situ* measurements of methanol in seawater have revealed nanomolar standing stocks and a methanol turnover time of 1 day (8), suggesting that ambient methanol concentrations in seawater could support significant microbial growth. To examine the extent to which the carbon amendments in our experiments directly supported the observed cell yields, we developed a simple, high-performance liquid chromatography (HPLC)-based method to quantify methanol in our seawater medium and the carbon-amended treatments.

**FIG 2:**
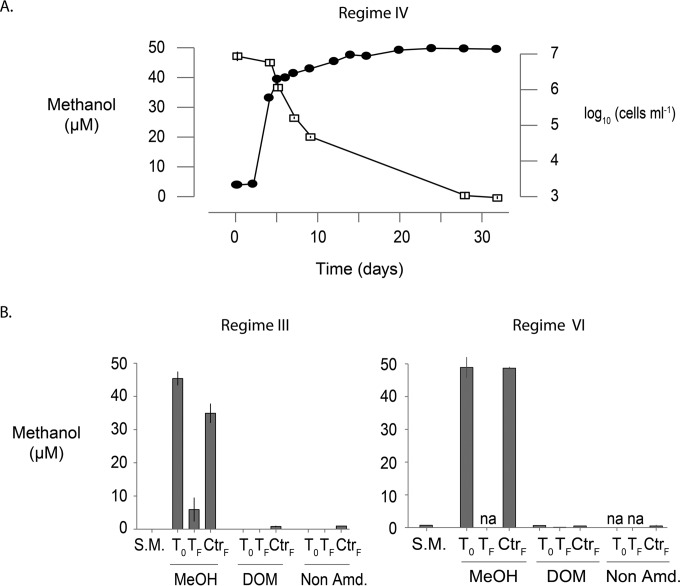
Methanol concentrations in Sargasso seawater medium with or without strain NB0046 and with different nutrient regimes and carbon substrates. (A) Time series of methanol drawdown in proportion to NB0046 growth in the nutrient- and vitamin-amended regime (IV). Cell densities are black filled circles, and methanol concentrations are open boxes. (B) Beginning and endpoint methanol concentrations for the nutrient- and vitamin-amended regime (III) and the UV-oxidized medium experiment with HMW DOM plus nutrient amendments (regime VI). The carbon substrate treatments are indicated below the axis (Non Amd., not carbon amended), and above those are the time points sampled (S.M., seawater medium before starting the experiment; T_0_, inoculated and just after addition of the carbon substrate; T_F_, inoculated and at the final time point sampled; Ctrl_F_, noninoculated at the final time point sampled). na, sample not available for analysis. Error bars for all plots are SDs of biological triplicates.

The ambient methanol concentration in the basal seawater was 248 ± 100 nM (mean ± standard deviation [SD], *n* = 6), comparable to mass spectrometry measurements of methanol in seawater from similar Atlantic latitudes by Beale et al. (8). On the basis of the bacterial growth efficiency of 22% reported by Halsey et al. (16) for OM43 strain HTCC2181 grown on 10 µM methanol and a bacterial carbon content of 10 fg of C cell^−1^ (28), this background methanol concentration could support a cell density of ca. 6 × 10^4^ ml^−1^ but not the 3 × 10^5^ to 5 × 10^5^ ml^−1^ we observed in the non-carbon-amended and HMW DOM treatments (Fig. 1). For the methanol-amended samples in regime IV, NB0046 consumed methanol at a rate inversely proportional to cell growth (Fig. 2A), and assuming the aforementioned growth efficiencies, the 46.6 µM methanol consumed could support the production of 1.2 × 10^7^ cells, comparable to the observed maximum cell density of 1.4 × 10^7^ ± 1.1 × 10^6^ ml^−1^ (mean ± SD, *n* = 3). A similar result was obtain under the methanol amendment in regime III (Fig. 2B), with a final observed methanol drawdown of 39 µM that could theoretically support a density of 1 × 10^7^ cells ml^−1^, closely matching the observed maximum cell density of 1 × 10^7^ ± 1.3 × 10^6^ ml^−1^ (mean ± SD, *n* = 3). Unexpectedly, there was also a small but significant abiotic loss of methanol in the noninoculated controls in the methanol treatment in this experiment, potentially due to the ventilation and shaking conditions of this regime (if this loss was consistent, the total drawdown would be reduced to 29 µM and the theoretical yield would be 0.74 × 10^7^ cells ml^−1^).

In the HMW DOM treatments, there were no significant increases in methanol concentrations just after the HMW DOM addition or at the final time point for the HMW DOM-amended, noninoculated controls (Fig. 2B). In the UV-oxidized seawater experiments in which we observed significant growth on HMW DOM, the methanol concentration was initially 0.67 ± 0.05 µM (mean ± SD, *n* = 3), 3.5 times as high as that of non-UV-treated seawater medium (Fig. 2B). This increase may be due to the UV-driven release of methanol from background HMW DOM polysaccharides naturally present in the seawater, which incorporate methylated sugars (29, 30). However, under the aforementioned assumptions, this would theoretically support only one-third of the observed final cell yields. Furthermore, the cells did not grow in either the non-carbon-amended control or methanol-amended treatment in the UV-oxidized seawater (Fig. 1), even given the medium’s higher background methanol concentrations. We did observe a drawdown of 0.54 µM methanol from the background medium at the end of the experiment for the inoculated HMW DOM treatment in UV-oxidized seawater, which could support 1.4 × 10^5^ cells ml^−1^ but not the observed final yield of 5.4 × 10^5^ cells ml^−1^. These results suggest that the addition of HMW DOM to the medium provided some growth factor or carbon source to support the observed cell production. It is unlikely that the growth of OM43 cultures on HMW DOM is due to abiotic release of absorbed or covalently attached methyl compounds from the DOM polymer, as we did not observe the presence of free methanol, formaldehyde, or formic acid in solutions of HMW DOM screened by ^1^H nuclear magnetic resonance (NMR) analysis, and there was no significant increase in the methanol concentration just after HMW DOM addition or at the final time point in the HMW DOM-amended, noninoculated controls (Fig. 2B). These results suggest that abiotic release of methanol from HMW DOM is negligible and support the hypothesis that HMW DOM-sustained growth is due to enzymatic cleavage of the carbon substrate directly from HMW DOM by the methylotrophs.

### Sequence composition and internal standard recovery.

**FIG 3:**
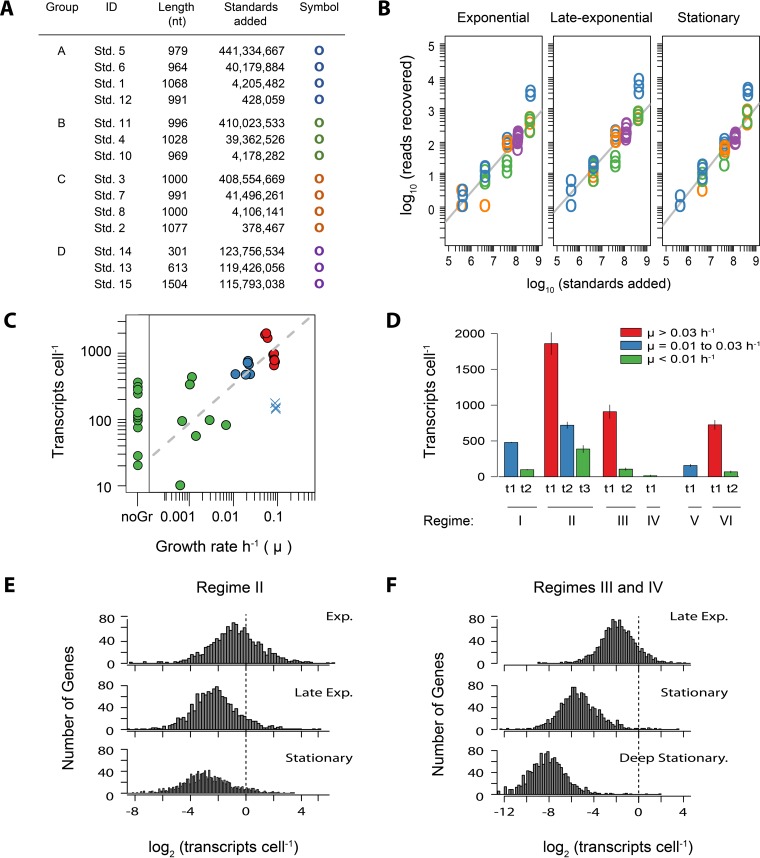
(A) Internal RNA standard statistics and groupings. ID, the standard identifier; length, total number of nucleotides in a standard; standards added, the final number of standards added to each sample; symbol, the plotting symbol in the graph. (B) The recovery of internal standards in the sequence libraries versus the number of standards added for samples collected over three different growth phases in the nutrient- and methanol-amended regime (II). The grey line is the fitted linear regression. Plots of standard recovery for all 33 samples are provided in Fig. S2. (C) Relationship between the total mRNA content per cell as estimated from internal standard recovery and the culture growth rate at the time of sampling. Points are colored as indicated in the legend to panel D. The time points at which growth was zero or cell densities were decreasing were binned into “noGr.” Regime V (UV-oxidized medium not nutrient amended plus HMW DOM) had relatively low temporal resolution of cell concentrations just before transcriptome sampling, causing the sampling time points to have an overestimate of the growth rate and were therefore considered outliers (plotted as blue ×s). (D) Total cellular transcript abundances by experiment and sampling time point (t1 to t3), as shown by the arrows in Fig. 1. (E, F) Distribution of transcript abundances in OM43 strain NB0046 during different phases of growth. The dashed line indicates the number of genes with one transcript cell^−1^. Exp., exponential growth phase; late exp., late-exponential growth phase; Deep Stationary., cultures in the stationary phase for an extended period of time.

Triplicate biological samples for RNA analysis were collected at 11 time points in the six cultivation experiments (Fig. 1) in order to identify transcriptional markers of growth, activity, and carbon metabolism under the different nutrient regimes. Quantitative transcriptomics was achieved by synthesizing 14 RNA standards that were assembled into four groups and then individually added to a sample just prior to RNA extraction (Fig. 3A; also see the supplemental material). The samples were rRNA depleted and sequenced with Illumina’s MiSeq platform. The sequencing yields across the 33 transcriptomes ranged from 3 × 10^5^ to 3 × 10^6^ reads per sample (see Table S1 in the supplemental material). Recovery of the internal RNA standards in the sequence libraries was log linear over 4 orders of magnitude, showing good replication between the standard sets and few indications of systematic or technical error (Fig. 3B; also see Fig. S2 in the supplemental material). However, 3 of the 14 standards were not recovered at the expected ratios (standards 2, 4, and 5; Fig. 3B; see Text S1 in the supplemental material) and therefore were not included in any downstream calculations. The remaining 11 standards were used to convert the number of reads per library to the number of transcripts per cell (see Text S2).

### Cellular mRNA content and transcript abundance as functions of the growth phase.

The average mRNA content per cell varied by 3 orders of magnitude across the six experiments and significantly correlated with the culture growth phase (Pearson’s *r* = −0.55, *P* < 0.01; Fig. 3C). The nutrient-amended regime (II) plus methanol, for which transcriptome samples were collected at three distinct points in the growth curve, had abundances of 1,859 (± 155), 720 (± 45), and 387 (± 50) transcripts cell^−1^ during the exponential, late-exponential (i.e., transitioning from exponential to stationary), and stationary growth phases, respectively (mean ± SD). The mRNA content of cultures in stationary phase for an extended period of time was lower (<100 transcripts cell^−1^) and reached a minimum of 17 ± 7 transcripts cell^−1^ in the 600th hour of the regime IV experiment (Fig. 3D).

Transcripts were detected for >99% of the genes in the NB0046 genome, no matter the experiment or treatment condition (99.8% ± 0.17% [mean ± SD], *n* = 33). The genome-wide distribution of gene-specific transcript abundances followed a lognormal distribution (Fig. 3E and F). These results are comparable to earlier RNA-Seq observations that showed that most, if not all, genes are expressed in the bacterial genome, and these expression levels follow a continuous distribution, with no discrete division into low or high gene expression (31). The variance in gene-specific transcript abundances was tightly constrained, with the difference between the lowest and highest gene expression levels spanning 5 orders of magnitude for all of the samples (4.6 ± 0.4 [mean ± SD], *n* = 33; Fig. 3E and F). In contrast, the magnitude of individual transcript abundances showed clear trends of shifting with the culture growth phase (Fig. 3E and F), as expected given the total cellular mRNA content relationship to the growth phase described above. The gene-specific transcript abundance averaged 1.4 ± 0.1 transcripts cell^−1^ during exponential phase, a value similar to that obtained by Passalacqua et al. (31), who used a modeling approach based on samples of *Bacillus anthracis* collected in exponential growth phase. The most highly expressed gene in the NB0046 transcriptome during this growth phase was the methanol dehydrogenase-encoding gene *xoxF* (NB46_00364), with a mean abundance of 83 ± 51 transcripts cell^−1^, while in contrast, the least expressed genes were found at only 1 transcript in every 215 cells (0.005 ± 0.002 transcripts cell^−1^). As the cultures entered stationary phase, the average gene transcription level dropped by 62% to 0.55 ± 0.03 transcripts cell^−1^ and eventually to a low of 0.29 ± 0.04 transcripts cell^−1^ in stationary phase. On the extreme end, in the deep stationary phase of regime IV, the average transcript abundance was 0.004 transcripts cell^−1^ or approximately 1 in every 250 cells. For the cellular transcript abundances of all of NB0046’s genes across the 33 different samples, see Table S2 in the supplemental material.

### Interpretation of cellular transcript abundances is growth phase dependent.

**FIG 4:**
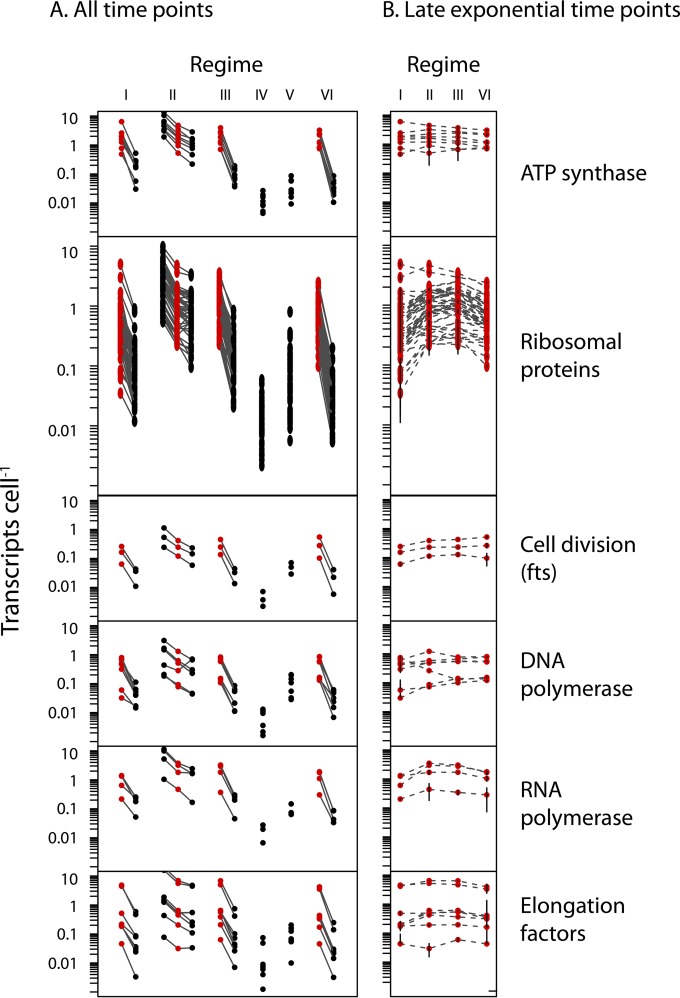
NB0046 transcription of genes indicative of growth, activity, or replication. (A) Gene-specific cellular transcript abundances at all sampling time points. Red points represent samples originating from cultures in the late exponential phase of growth. (B) Transcript abundances of only the late-exponential-phase samples indicate that samples in similar growth phases tended to have similar transcript abundances.

An examination of genes commonly used as metabolic state and growth phase markers revealed that the cellular transcript abundance of these genes closely tracks the growth status of the cultures at the time of collection (Fig. 4A). Given that the goal of most transcriptome experiments is a comparative analysis of treatment-specific processes, these results suggest that comparisons of different treatments on a transcripts-per-cell scale should be conducted with sample populations in similar growth/activity states, or else global transcript changes tied to the physiological state may obscure any biologically meaningful difference between the treatments. Therefore, in our study, to identify treatment-specific differences in transcript abundances, we compared only across time points when the cultures were in similar growth phases, particularly focusing on the four experiments that had samples near the end of exponential-phase growth, i.e., methanol (MeOH)-amended medium without inorganic nutrients (N) or vitamins (V) (regime I, +MeOH −N −V); methanol- and inorganic-nutrient-amended medium (regime II, +MeOH +N −V); methanol-, inorganic-nutrient-, and vitamin-amended medium (regime III, +MeOH +N +V); and HMW DOM- and inorganic-nutrient-amended, UV-oxidized medium (regime VI, +DOM +N −V).

While cell concentrations differed greatly among these samples (Fig. 1), a statistical analysis of growth- and activity-related genes revealed significantly similar cellular transcript abundances among these samples (Fig. 4B; analysis of variance [ANOVA], Benjamini-Hochberg-corrected *P* < 0.05; log_2_-fold change, <1).

### Central carbon metabolism.

**FIG 5:**
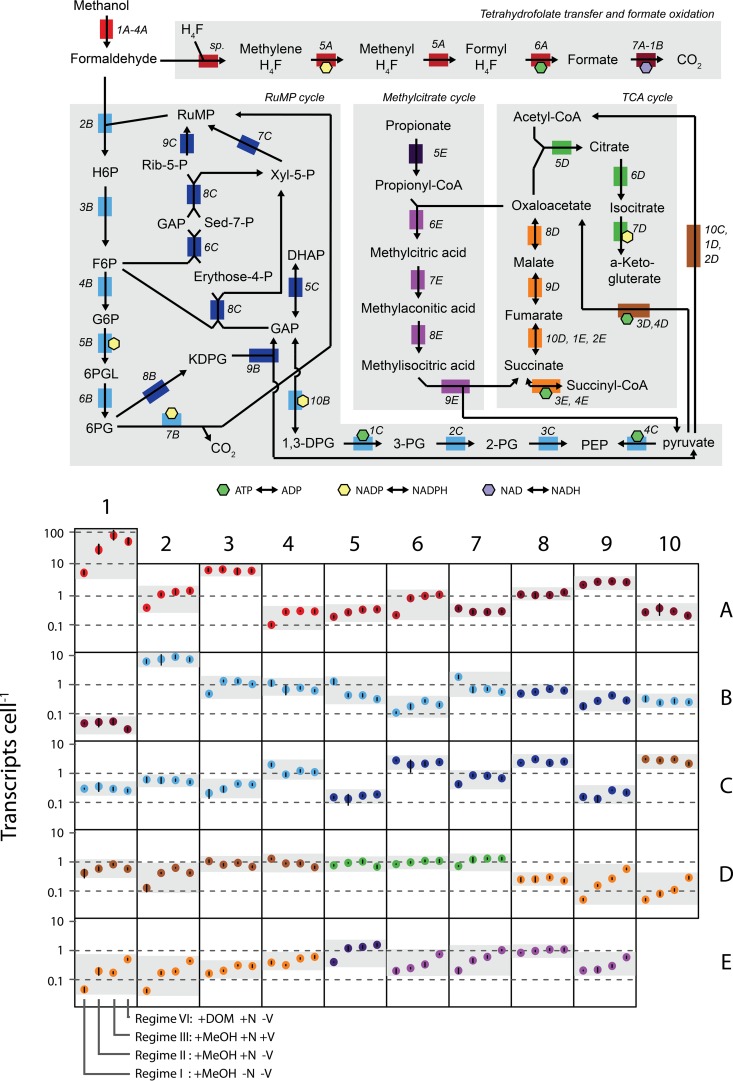
Transcript abundances of NB0046 genes related to one-carbon and central metabolism under different nutrient regimes and carbon substrate additions. At the top is a metabolic map of the substrates, genes (colored blocks), and their connections (arrows) in strain NB0046. Next to each gene is an identifier in italics providing the location of that gene in the bottom panel, which shows the mean transcript abundances of triplicate samples collected from the late exponential phase for the different nutrient regimes (regime designations are shown at the base of box 1E). The full names and IMG accession numbers of the genes are shown in Table S3 in the supplemental material. Error bars are the SDs of biological triplicates.

In order to identify regulatory patterns of key metabolic processes, we explored the NB0046 transcript abundances of genes involved in C1 and central carbon metabolism sampled from the same growth phase (the four late-exponential-phase experiments listed above) but with various nutrient regimes and carbon substrates (Fig. 5). Genes that had a significant difference across treatments were identified by ANOVA, and if the Benjamini-Hochberg-corrected *P* value was <0.05, a pairwise *t* test was used to identify significantly different treatment pairs with a minimum 2-fold difference in transcript abundance.

The initial step in C1 metabolism is methanol oxidation to formaldehyde via an *xoxF*-type methanol dehydrogenase (32, 33), and in our experiments, *xoxF* was often the most highly transcribed gene, with tens to hundreds of transcripts cell^−1^ (Fig. 5, box 1A). This is consistent with observations from coastal transcriptomes that *xoxF* is often a dominant component of the marine transcript and protein pool (20, 22). In fact, under all of the conditions tested in this study, *xoxF* was always present at ≥1 copy cell^−1^, even in stationary phase. This was true of no other gene in the genome, further highlighting *xoxF*’s likely vital function or rapid cellular turnover. Interestingly, while methanol was present at nanomolar or greater concentrations in all of our experiments (Fig. 2), *xoxF*’s transcriptional dynamics seemed to be more nutrient regime dependent, with a 20-fold difference in *xoxF* transcript abundance between the nutrient-deplete (+MeOH −N −V) and nutrient-amended (+MeOH +N −V) regimes (Fig. 5, box 1A). Genes *xoxJG* are also part of the *xox* operon but were transcribed at lower levels than *xoxF* and displayed more muted expression dynamics, with *xoxJ* (methanol dehydrogenase-associated protein) showing significantly lower expression only in the nutrient-deplete experiment (+MeOH −N −V) and *xoxG* (associated cytochrome) consistently expressed at 10 to 11 transcripts cell^−1^ across all four experiments (Fig. 5, boxes 2A and 3A).

Formaldehyde produced from methanol oxidation is the central intermediate in C1 metabolism, and OM43 strains possess two different pathways for processing it: (i) oxidation of formaldehyde to formate via the tetrahydrofolate pathway and (ii) the ribulose monophosphate (RuMP) cycle for either assimilatory or dissimilatory processes. The first two enzymatic steps in the tetrahydrofolate pathway are catalyzed by a bifunctional *folD*-encoded protein, which, in all of our late-exponential-phase experiments, was consistently expressed at ca. 0.3 transcript cell^−1^ (Fig. 5, box 5A). The methyl moiety is then oxidized to formate via a formate-tetrahydrofolate ligase, which showed significantly lower expression in the nutrient-deplete regime (+MeOH −N −V) (Fig. 5, box 6A). The final step in the tetrahydrofolate pathway is oxidation of formate to CO_2_ via a multisubunit formate dehydrogenase, which was relatively consistently transcribed across the various nutrient regimes, although the transcript abundances of the individual subunits were substantially different from one another (averages of 0.05, 0.31, 1.16, and 2.94 transcripts cell^−1^ for the delta, gamma, beta, and alpha subunits, respectively) (Fig. 5, boxes 7A through 1B).

An alternative route for formaldehyde metabolism is the RuMP cycle, which can operate in an assimilatory manner to produce intermediates of central carbon metabolism or in a dissimilatory manner for energy conservation. Entry into the RuMP cycle begins with 3-hexulose-6-phosphate synthase (HPS), which condenses formaldehyde and ribulose 5-phosphate. We observed that HPS had consistently high late-exponential-phase transcript abundances under all of the nutrient regimes (8 to 12 transcripts cell^−1^; Fig. 5, box 2B). After HPS, RuMP intermediates undergo a series of isomerizations and dehydrations (Fig. 5, boxes 3B and 6B). Interestingly, the genes encoding dehydration steps were two of the few genes in the genome we observed to have significantly higher transcript abundances under nutrient-deplete conditions (+MeOH −N −V) (Fig. 5, boxes 5B and 7B). These two dehydrogenases are important regulatory steps in the RuMP cycle (34), with 6-phosphogluconate dehydrogenase in particular being diagnostic of the dissimilatory portion of the RuMP cycle, which leads to the oxidation of phosphogluconate to CO_2_ and the regeneration of RuMP. The transcriptional enrichment of these dehydrogenases may indicate that the cells were routing more carbon to the dissimilatory component of the RuMP cycle under nutrient-deplete conditions, potentially due to a lack of key inorganic nutrients for synthesizing biomass.

After the assimilatory C1 and RuMP pathways, carbon may enter the TCA cycle, which, in all sequenced OM43 strains, is missing the E1 subunit of α-ketoglutarate dehydrogenase (15, 17). This enzymatic gap results in an incomplete TCA cycle, which is widely used as a diagnostic marker of obligate methylotrophy (35). In our experiments, the two TCA cycle branches displayed distinctly different transcriptional patterns. The first branch consists of the conversion of citrate to α-ketoglutarate, and the three genes in this branch were consistently transcribed at ca. 1 transcript cell^−1^ across all of the regimes (Fig. 5, boxes 5D through 7D). The second TCA cycle branch is the conversion of oxaloacetate to succinate, and interestingly, it showed an expression pattern that was very different from that of the citrate branch, ranging from a low of 0.1 transcript cell^−1^ in the nutrient-deplete cultures (+MeOH −N −V) to ca. 1.0 transcript cell^−1^ in the HMW DOM treatment (+DOM +N −V) (Fig. 5, boxes 9D and 2E). The NB0046 genome encodes a succinate dehydrogenase, and although it was also significantly enriched in the HMW DOM treatment, the effect size was much smaller than that of the other TCA genes (Fig. 5, boxes 3E and 4E), potentially indicating a different metabolic role, as it is unknown how the conversion of succinate to succinyl-CoA would integrate into central metabolism in these organisms (35). Similarly, many members of the family *Methylophilaceae* do not encode a canonical malate dehydrogenase, instead encoding a malate quinone oxidoreductase (Fig. 5, box 8D; see reference 35). Notably, in our experiments, the gene for this enzyme did not follow the same transcription pattern as the other genes, potentially suggesting a different role or an alternative enzyme in this location.

Several genes associated with methylcitrate metabolism had transcription patterns similar to that of the oxaloacetate arm of the TCA cycle. An examination of the NB0046 genome revealed that these genes are likely part of a complete methylcitrate cycle (MCC). In other organisms, the MCC has been shown to be involved in the metabolism of short-chain fatty acids, particularly propionyl coenzyme A (propionyl-CoA) (36). In NB0046, MCC genes for propionyl-CoA metabolism are clustered together in a *prpBCD* locus encoding 2-methylcitrate synthase (*prpC*; Fig. 5, box 6E), 2-methylcitrate dehydratase (*prpD*; Fig. 5, box 7E), and methylisocitrate lyase (*prpB*, a diagnostic marker of the MCC pathway; Fig. 5, box 9E). Strain NB0046 does not appear to encode a canonical 2-methylisocitrate dehydratase, though it does contain an aconitate hydratase (*acnB*, Fig. 5, box 8E), which may have similar activity. A survey of OM43 clade genomes in the Integrated Microbial Genomes (IMG) database revealed the *prpBCD* locus to be conserved among all four available genomes (HTCC2281, HIMB624, NB0016, NB0046).

The MCC is completed by the oxaloacetate branch of the TCA cycle, and the transcription patterns of these two cycles were highly similar. Just as the oxaloacetate TCA branch transcripts were significantly enriched in the HMW DOM treatment (+DOM +N −V), we also observed significantly higher MCC transcript abundances in the HMW DOM samples (Fig. 5, boxes 6E, 7E, and 9E). The one exception was the aconitate hydratase gene mentioned above (*acnB*, Fig. 5, box 6E), which may suggest that NB0046 encodes the MCC-2 pathway variant that does not require this enzyme.

The MCC has also been found in *Methylotenera mobilis*, a related member of the family *Methylophilaceae* from freshwater sediments, suggesting that they have the capability for multicarbon metabolism and challenging the notion that these organisms are obligate methylotrophs (37). Expression of *M. mobilis* MCC genes was enriched *in situ*, suggesting that 3C metabolism may be an important component of their *in situ* activity. Our observations of a complete MCC cycle in marine members of the OM43 clade indicates that these organisms may have the ability to degrade multicarbon substrates, and the upregulation of these genes in the HMW DOM treatment suggests a potential route for metabolizing this complex carbon substrate.

### Transcriptional responses to differing nutrient regimes.

Beyond central carbon metabolism, NB0046 also had substantial transcriptional shifts in other important physiological processes under the different nutrient regimes, including those related to inorganic nutrient processing and energy conservation (Table 1), as well as translational and iron-related machinery (see Text S1 in the supplemental material).

**TABLE 1:** Cellular transcript abundances of selected genes significantly upregulated or downregulated under different nutrient regimes^a^

No. of transcripts per 1,000 cells				Gene description	Locus	SD; significance
Deplete	Replete	Vitamin	DOM			
17,897	10	10	8	Conserved hypothetical protein	NB46_00251	166/3/3/2; +++, −nn, −nn, −nn
3,093	3	3	3	Nitrogen regulatory protein P_II_	NB46_00250	86/1/1/1; +++, −nn, −nn, −nn
4,952	18	19	23	Ammonium transporter	NB46_00249	1,402/3/4/2; +++, −nn, −nn, −nn
32	2	1	1	Hypothetical protein KB13_177	NB46_00540	19/2/1/1; +++, −nn, −nn, −nn
425	35	69	83	Ammonium transporter	NB46_01065	119/2/14/8; +++, −nn, −nn, −nn
2,413	2,058	1,899	1,653	Glutamine synthetase, type I	NB46_00305	651/287/315/162; +++, −nn, −nn, −nn
1,228	236	379	370	Glycosyltransferase involved in cell wall biogenesis	NB46_00223	244/41/36/42; +++, −nn, −nn, −nn
869	177	184	173	Nitrogen regulation protein NtrB	NB46_00304	70/20/27/30; +++, −nn, −nn, −nn
2,826	565	1,162	936	IMP dehydrogenase	NB46_01283	792/108/135/90; +++, −nn, −nn, −nn
1,277	319	558	415	GMP synthase	NB46_01284	152/16/20/38; +++, −−n, −+n, −nn
88	994	446	175	Chaperone protein DnaJ	NB46_00532	6/473/30/3; −nn, +n+, nnn, n−n
69	889	363	165	Cochaperone GrpE	NB46_00534	8/496/73/14; −nn, +n+, nnn, n−n
127	2,066	1,064	493	Chaperone protein HtpG	NB46_00999	12/673/182/38; −−n, +++, ++n, n−n
372	4,704	2,003	944	Chaperone protein DnaK	NB46_00533	29/829/313/71; —n, +++, +−+, n−−
563	2,833	946	547	Chaperonin GroS	NB46_01193	43/543/208/15; −nn, +++, n−n, n−n
1,240	6,068	2,213	1,305	Chaperonin GroL	NB46_01192	167/681/325/55; —n, +++, −+−, n−−
570	750	718	560	Phosphate transport system regulatory PhoU	NB46_00097	75/34/32/50; —n, +n+, +n+, n−−
76	109	163	183	Phosphate-selective porins O and P	NB46_00625	28/34/22/32; n−−, nnn, +nn, +nn
40	83	119	162	Phosphate ABC transporter, periplasmic P-binding	NB46_00628	7/8/14/19; −−−, +−−, ++−, +++
154	240	453	439	Phosphate ABC transporter, permease PstC	NB46_00629	12/30/12/59; −−−, +−−, ++n, ++n
95	210	531	493	Phosphate ABC transporter, permease PstA	NB46_00630	9/29/20/49; −−−, +−−, ++n, ++n
94	280	457	361	Phosphate ABC transporter, ATP-binding	NB46_00631	20/52/32/16; −−−, +−−, +++, ++−
78	183	202	176	Phosphate regulon sensor protein	NB46_01228	8/99/6/10; nnn, nnn, nnn, nnn
134	264	1,213	989	PhnP protein	NB46_00720	8/53/78/99; −−−, +−−, +++, ++−
308	644	298	244	Fe-S protein assembly chaperone HscA	NB46_00974	42/268/9/24; nnn, nn+, nnn, n−n
109	155	87	88	Fe-S protein assembly cochaperone HscB	NB46_00975	15/45/13/7; nnn, nnn, nnn, nnn
170	350	163	230	Iron-sulfur cluster assembly protein IscA	NB46_00976	16/26/27/30; −n−, +++, n−−, −+−
134	5,304	574	309	TonB-dependent siderophore receptor	NB46_01062	8/1,302/38/44; −nn, +++, n−n, n−n
289	1,148	301	964	Putative TonB-dependent receptor	NB46_00103	15/321/22/71; −n, ++n, n−−, +n+
1,376	688	772	671	Ferritin and Dps	NB46_00108	79/103/47/48; +++, −nn, −nn, −nn
1,455	16,208	36,814	27,421	Bacteriorhodopsin	NB46_00176	121/1,449/4,097/3,176; −−−, +−−, +++, ++−
39	72	95	76	β-Carotene 15,15′-monooxygenase	NB46_00171	1/38/4/8; n−n, nnn, +nn, nnn
27	139	151	158	Lycopene cyclase protein	NB46_00172	2/67/19/15; −−−, +nn, +nn, +nn
22	106	93	113	Phytoene/squalene synthetase	NB46_00173	4/36/13/16; −−−, +nn, +nn, +nn
101	304	329	361	Phytoene desaturase	NB46_00174	21/129/34/43; −−−, +nn, +nn, +nn
147	522	630	673	Geranylgeranyl pyrophosphate synthase	NB46_00175	27/154/152/92; −−−, +nn, +nn, +nn
44	47	102	94	2-C-methyl-d-erythritol 2,4-cyclodiphosphate	NB46_01067	12/8/2/6; n−−, n−−, ++n, ++n
50	35	114	103	2-C-methyl-d-erythritol 4-phosphate transferase	NB46_01066	8/5/12/10; n−−, n−−, ++n, ++n
98	260	687	666	Peptidase PpqF	NB46_00526	24/58/38/72; −−−, +−−, ++n, ++n
180	379	863	750	Putative Xaa-Pro aminopeptidase 3	NB46_00896	14/111/75/74; −−−, +−−, ++n, ++n
66	234	715	683	Trypsin domain protein	NB46_01139	20/44/24/95; −−−, +−−, ++n, ++n
84	151	437	507	ABC-type dipeptide transport system, ATPase	NB46_00835	3/35/23/94; n−−, n−−, ++n, ++n
85	239	553	385	Peptide ABC transporter, permease protein	NB46_00784	10/127/21/34; −−−, +−−, +++, ++−
92	314	706	757	Sigma E regulatory protein, MucB/RseB, putative	NB46_00988	13/67/42/97; −−−, +−−, ++n, ++n
386	1,211	2,478	2,690	RNA polymerase sigma factor RpoE	NB46_00986	103/196/256/333; −−−, +−−, ++n, ++n
66	51	139	113	Sulfatase	NB46_00552	8/12/10/13; n−−, n−−, +++, ++−
121	230	648	511	Extracellular solute-binding protein, family 5	NB46_00783	5/83/46/71; n−−, n+−, +++, ++−
16	77	66	193	Hypothetical protein Neut_0862	NB46_00664	0/3/4/4; −−−, ++−, +−−, +++
12	30	28	70	FKBP-type peptidyl-prolyl *cis*-*trans* isomerase	NB46_01197	4/5/7/15; nn−, nn−, nn−, +++
101	269	420	1,055	Putative HpcH/HpaI aldolase/citrate lyase	NB46_00665	12/33/16/47; −−−, +−−, ++−, +++
104	341	487	1,045	Long-chain fatty acid–CoA ligase, putative	NB46_00666	17/93/21/76; −−−, +−−, ++−, +++

a Abundances are the mean values of triplicates with the SDs in the far right column. Deplete, +MeOH −N −V; replete, +MeOH +N −V; vitamin, +MeOH +N +V; DOM, +DOM +N −V. Statistically significant upregulation (+) or downregulation (−) (ANOVA and *t* test; *P* < 0.05) by a treatment against the three other treatments is indicated in the far right column for each of the conditions (n, no significant difference) in the following order: deplete, replete, vitamin, and DOM. Table S2 in the supplemental material contains the cellular transcript abundances of all of the genes in NB0046.

### Nitrogen.

Under the nutrient-deplete regime (+MeOH −N −V), NB0046 substantially upregulated nitrogen-related transcripts, particularly those involved in the P_II_-dependent response, the glutamine oxoglutarate aminotransferase (GOGAT) system, and nitrogen starvation stress (Table 1). This included genes such as that for the P_II_ nitrogen regulatory protein (>1,000-fold increase in transcript abundance), that for glutamine synthase (6-fold increase), and *ntrB* (5-fold increase). NB0046’s two ammonium transporters also had higher cellular transcript abundances in the nutrient-deplete experiment, although to a markedly different extent, with *amtB* gene 1 (NB46_01065) increasing 2-fold (0.4 ± 0.1 transcript cell^−1^ [mean ± SD]), while *amtB* gene 2 (NB46_00249) increased over 250-fold to 4.9 ± 1.4 transcripts cell^−1^. Other nitrogen-related transcripts enriched in the nutrient-deplete condition included those for IMP dehydrogenase, GMP synthase, ornithine carbamyl transferase, and carbamyl phosphate synthase (Table 1). The gene showing the greatest change in cellular transcript abundance over all of the experiments was that for hypothetical protein NB46_00251, which is in a putative operon with the gene for the highly upregulated ammonium transporter and nitrogen P_II_ regulatory protein (Glnk). For the majority of experiments, this gene’s transcripts were typically found in only 1 out of every 100 cells; however, under the nutrient-deplete regime (+MeOH −N −V), the abundance of the transcript for NB46_00251 increased by 1,900-fold to 17 ± 0.2 transcripts cell^−1^(Table 1).

### Phosphorus.

Changes in phosphorus-related transcript abundances among the different nutrient conditions were more constrained than those for nitrogen-related transcripts (Table 1). The nutrient-deplete regime (+MeOH −N −V) had a significantly lower transcript abundance (2- to 5-fold) of the phosphate-specific transport system gene (*pst*), while that of the gene for the corresponding negative regulator protein, *phoU*, showed little difference among the experiments. The genes for the phosphate regulon sensor protein and a phosphate-selective porin were also significantly lower in the nutrient-deplete regime. While *pst* transcripts increased upon phosphorus addition (+MeOH +N −V), they reached their highest abundances in the vitamin (+MeOH +N +V) and HMW DOM regimes (+DOM +N −V). Interestingly, there was also significant enrichment in both the vitamin-plus-methanol and HMW DOM regimes of a *phnP* gene that encodes the last step in methylphosphonate utilization (Table 1). The NB0046 genome, however, does not appear to contain the other genes in the canonical carbon-phosphonate lyase operon, suggesting that this gene may have a different metabolic role in NB0046. *phnP* is a phosphoribosyl 1,2-cyclic phosphate phosphodiesterase that is able to cleave phosphodiesterase bonds. Cyanocobalamin (vitamin B_12_), which contains a phosphate group in a cyclic phosphodiesterase bond, is part of the AMS1 vitamin mixture used in our experiments and was present at a final concentration of 700 pM. In a similar fashion, chemical characterization of HMW DOM by ^31^P NMR shows that up to 70% of the organic phosphorus in HMW DOM is contained within sugar phosphodiester bonds (D. J. Repeta, unpublished data), such that the HMW DOM regime was supplemented with ~1.3 µM sugar phosphodiester-P. Thus, metabolism of cyanocobalamin and HMW DOM may be linked by the substrate-induced transcription of genes for breaking phosphodiesterase bonds (*phnP*) and phosphate uptake (*pst*).

### Rhodopsin.

The NB0046 genome encodes a light-driven, proton-pumping rhodopsin, and under the methanol- and nutrient-amended (+MeOH +N −V), methanol- and vitamin-amended (+MeOH +N +V), and HMW DOM (+DOM +N −V) conditions, cellular abundances of rhodopsin transcripts were high, with 16 to 36 transcripts cell^−1^ (Table 1). These findings agree with metatranscriptomic surveys in which rhodopsin genes are often found to be some of the most highly transcribed genes *in situ*, not just for OM43 clade members but for many abundant community members such as SAR11, SAR116, and flavobacteria (21, 38). However, under nutrient-deplete conditions (+MeOH −N −V), rhodopsin transcripts were significantly reduced by 18-fold to 1.5 ± 0.1 transcripts cell^−1^ (mean ± SD). Rhodopsin-associated genes were also significantly reduced under these nutrient-deplete conditions, including carotenoid biosynthesis and β-carotene monooxygenase genes, which encode the final step in retinal synthesis (Table 1). These results show that rhodopsin is not simply constitutively transcribed in an OM43 clade representative and match recent metatranscriptomic and quantitative PCR studies that show modulation of rhodopsin transcription *in situ*, particularly on diel time scales (39, 40). While the physiological drivers of these modulations in the environment are not well understood, studies of several marine isolates have shown that organisms may use proteorhodopsin-derived energy to survive periods of starvation (41). Steindler et al. (42) demonstrated for a streamlined bacterium of the SAR11 clade that rhodopsin-driven ATP production was energetically advantageous when it was carbon starved, but provided no detectable advantage during active growth on organic carbon substrates. This may explain NB0046’s downregulation of rhodopsin transcripts under nutrient-deplete conditions (+MeOH −N −V), as inorganic nutrients were likely limiting growth while simultaneously there was an abundant carbon source (methanol) for energy conservation, thus negating the need for the supplemental energy source that rhodopsin-driven proton pumping can provide.

### Global similarity between vitamin-amended and HMW DOM treatments.

While there were clear instances of transcription differences between the vitamin-plus-methanol (+MeOH +N +V) and HMW DOM (+DOM +N −V) regimes, the two experiments often displayed similar expression patterns (Table 1), including enrichment of genes for the nonmevalonate pathway for terpenoid synthesis (which integrates with synthesis of vitamins B_1_ and B_6_, carotenoids, and quinones), peptidases encoded by *ppqF* and *pqqG* (involved in the biosynthesis of pyrroloquinoline quinone), several genes for the uptake and metabolism of proteins (sulfatases, peptide ABC transport), and the phosphorus-related *phnP* gene described above. Notably, many of these genes can be linked to vitamin metabolism or to accessing functional groups on vitamin molecules.

**FIG 6:**
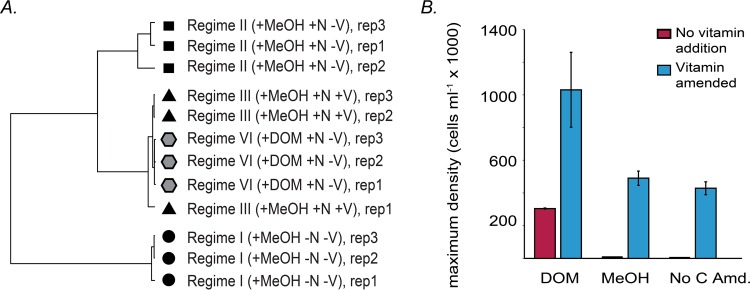
OM43 strain NB0046 transcription patterns and cell growth assays reveal similar responses to the vitamin (+MeOH +N +V) and HMW DOM (+DOM +N −V) regimes. (A) Hierarchical clustering (with Pearson correlation coefficients and complete linkage) of genome-wide transcript abundances. (B) Maximum NB0046 cell densities in UV-oxidized seawater medium with or without AMS1 vitamin mixture amendment after 72 h of incubation. Error bars are the SDs of biological triplicates. No C Amd., non-carbon-amended control.

To quantify the extent of the transcriptional similarity between the vitamin-plus-methanol and HMW DOM regimes further, we used hierarchal clustering to examine how the late-exponential-phase time points from each nutrient regime were related on a global transcriptome scale (Fig. 6A). While the nutrient-deplete (+MeOH −N −V) and nutrient-amended (+MeOH +N −V) regimes formed separate, distinct clusters, the vitamin-amended (+MeOH +N +V) and HMW DOM (+DOM +N −V) regimes clustered together, with the three HMW DOM replicates nested within the vitamin replicates (Fig. 6A). This strong similarity in the global transcriptome indicates that the HMW DOM additions provide vitamins or substituting nutrients that (at least in part) stimulate growth in UV-oxidized seawater. To test this, we conducted an experiment in which vitamins were added to UV-oxidized seawater medium amended with nutrients and looked for recovered growth in the methanol- and non-carbon-amended treatments. In comparison to non-vitamin-amended UV-oxidized seawater, the addition of vitamins stimulated NB0046’s growth in UV-oxidized seawater for both the methanol- and non-carbon-amended treatments (Fig. 6B). This was true for cells grown either in cultivation plates at 1 ml well^−1^ (Fig. 6B) or in 8-ml cultures in test tubes (data not shown). However, in the test tubes, we continued to see that methanol addition led to a substantial increase in cell concentrations over the HMW DOM addition when vitamins were added, in contrast to the cultivation plates in which the addition of vitamins to the UV-oxidized seawater led to maximum cell density for the HMW DOM amendments.

It is important to note that vitamin B_12_ (cobalamin) has a size of >1 kDa and is potentially captured at ~98 to 99% efficiency in the HMW DOM collection process (Repeta, unpublished). This may provide a partial explanation for the complex growth patterns we saw with HMW DOM. The purpose of UV oxidizing the seawater used was to reduce the concentration of background carbon compounds present in natural seawater that sustain the ca. 5 × 10^5^ cell ml^−1^ yields in non-carbon-amended experiments. Vitamins and vitamin precursors are likely oxidized during this process, and therefore, the fact that HMW DOM additions recover the growth of NB0046 cultures in UV-oxidized medium suggests that HMW DOM may contain some of these missing growth factors. An alternative explanation is that NB0046 can use the vitamins as a growth substrate, as evidenced by the small difference between the non-carbon-amended and methanol treatments in UV-oxidized seawater (Fig. 6B).

Support for the hypothesis that restored growth in the DOM-amended, UV-oxidized medium is related to some vitamin supplementation thus includes the facts that (i) vitamin B_12_ (and probably other B vitamins) is likely present in the HMW DOM concentrate; (ii) most organic cofactors will likely have been destroyed during the UV oxidation process; (iii) hierarchical cluster analysis revealed a global similarity in the vitamin-amended (+MeOH +N +V) and HMW DOM (+DOM +N −V) transcriptomes (Fig. 6A), suggesting a common metabolic response under these two conditions; and (iv) most importantly, addition of vitamins to the UV-oxidized medium restored the methanol growth response (Fig. 6B).

### Summary.

In this study, we produced a fully quantitative transcriptome of a marine bacterium in order to characterize the global transcript pool and shifts in cellular transcript abundances in response to different growth phases, nutrient regimes, and carbon substrates.

The mRNA content of this streamlined marine bacterium closely matched both modeled and experimentally derived values for other bacteria and was substantially influenced by the growth phase of the cultures at the time of collection. In the exponential phase of growth, NB0046 had 900 to 1,800 transcripts cell^−1^, a value consistent with classical measurements of cell content based on bulk macromolecular composition. For example, an average *Escherichia coli* cell in exponential growth has been shown to contain 1,380 transcripts cell^−1^ (based on measurements of RNA mass [43]) to 1,800 transcripts cell^−1^ (based on single-cell analysis [44]). NB0046’s cellular mRNA content during later phases of growth was lower, averaging approximately 100 transcripts cell^−1^. These NB0046 stationary growth phase values are similar to the low values observed in the field, with several studies showing that an average marine bacterial community member will contain ca. 100 to 300 transcripts cell^−1^ (23, 24, 45). However, it is not clear how well the growth phases of batch cultures reflect the nature of bacterioplankton growth in the environment. Caution in interpreting these values is thus urged, and more research is needed to fully understand how representative batch cultures in stationary phase may mirror the low but potentially steady-state growth of *in situ* populations (46, 47).

The majority of genes were transcribed at <1 copy cell^−1^, results similar to previous models of bacterial transcription in which few genes have transcripts in every cell of a population, even during rapid growth (31, 48). We observed that cellular transcript abundances of genes encoding basic cellular maintenance and growth processes (ATP synthase, polymerases, elongation factors, etc.) were highly correlated with the growth phase of the culture and reflected a broader, system-wide trend of transcript abundance being directly related to the growth phase. These results suggest that inferences about differential transcription on a per-cell basis between experimental conditions should be made from cultures sampled in similar growth phases. By comparing only those samples that were collected near the end of exponential growth, we observed NB0046 to differentially regulate the transcript abundances of individual genes in response to different environmental conditions and substrates, including genes encoding carbon, nitrogen, phosphorus, iron, and energy conservation-related processes (Table 1; see Table S2 in the supplemental material).

With their small genome and small cell size, OM43 clade members are categorized as having a streamlined lifestyle, which is increasingly recognized as a prominent ecological strategy in the oceans (49). Similar to SAR11 clade organisms, representatives of the OM43 clade have relatively few means of transcriptional regulation (49), suggesting a reduced ability to respond to environmental dynamics, at least on the transcriptional level. However, the large shifts in transcript abundance observed in this study suggest that OM43 members are capable of inducing substantial changes in their transcriptomes in response to differing environmental conditions. For example, NB0046 increased the transcript abundances of nitrogen-related genes such as those for the P_II_ response, GOGAT, and ammonium transporters by hundreds to thousands of fold in the nutrient-deplete regime (+MeOH −N −V). In contrast, a recent coupled transcriptome-proteome study of SAR11 clade representative “*Candidatus* Pelagibacter ubique” revealed that this organism had little transcriptional response to nitrogen limitation, with many of its transcript changes limited to <3-fold between nitrogen-deplete and nitrogen-replete cultures (50). This difference between the transcriptional responses of SAR11 and OM43 is likely partially due to a lack of a P_II-_dependent regulon system in SAR11. Indeed, in *Dehalococcoides mccartyi*, which, like NB0046, contains a P_II_ regulon, a >50-fold increase in P_II_-related gene transcription was observed under nitrogen limitation, suggestion that the P_II_ regulon is inherently prone to large transcriptional dynamics (51). These results suggest that even though two types of organisms may have streamlined characteristics in common, there can be significant differences in the extent of their regulatory responses to changing environmental conditions.

A central goal of this study was to determine how an OM43 clade representative responds to different carbon substrate availability. Our measurements of methanol concentrations in seawater medium showed that methanol drawdown occurs at a rate proportional to cell growth and closely follows a model of growth efficiency similar to that described by Halsey et al. (16). However, our transcription results suggest a relatively complex regulatory scheme controlling methanol metabolism that depends on nutrient conditions. For example, *xoxF* (methanol dehydrogenase), despite always being the most highly transcribed gene, had significantly higher transcript abundances in the nutrient-amended regimes, suggesting that the cell may upregulate this crucial pathway when nutrient supplies allow methanol to be used for both energy generation and biosynthesis. Accordingly, key dissimilatory RuMP cycle transcripts were upregulated in the nutrient-deplete regime, suggesting that when inorganic nutrients are limiting, carbon is routed more toward dissimilatory energy conservation processes. In contrast, proteorhodopsin transcription was downregulated in the nutrient-deplete regime (+MeOH –N −V), perhaps reflecting a decreased requirement for this alternative energy source when there is ample carbon for respiration-driven energy conservation but growth is limited by inorganic nutrient availability (42). Interestingly, formate dehydrogenase and hexulose phosphate synthase gene transcription remained relatively constant across the experiments, suggesting that these genes, though both highly transcribed, might not be the most sensitive transcriptional markers, at least not under the conditions tested here.

Our transcription data also provided insights into an OM43 clade representative’s growth on HMW DOM. The significant enrichment of transcripts for the oxaloacetate arm of the TCA cycle in the HMW DOM treatment, together with the detection of complete MCC in NB0046 that was also significantly upregulated in the HMW DOM treatment, was suggestive of a potential route for HMW DOM metabolism through three-carbon compounds. In addition, the global similarity between the transcriptional responses to the vitamin-plus-methanol (+MeOH +N +V) and HMW DOM (+DOM +N −V) treatments suggests that HMW DOM might also provide some vitamin supplements missing from the organic stripped UV-oxidized seawater that allow for the production of important metabolites like quinones, vitamins, carotenoids, and terpenoids. This supplementation might be the result of enrichment of vitamins within the HMW DOM itself or, alternatively, from both compounds serving directly as a growth substrate, with a potential metabolic link due to the presence of organic phosphorus contained within phosphodiesterase bonds. Taken together, these results suggest several potential mechanisms for an expanded metabolic range in OM43 strain NB0046. Future tests of these hypotheses are needed to tease apart the complex factors influencing OM43 carbon metabolism, including examine growth on three-carbon compounds and vitamins, and their potential origin in the HMW DOM complex.

This study highlights the power of combining quantitative transcriptomics with comparative growth experiments on a model organism to identify transcriptional markers of physiological and metabolic activities in order to build transcript inventories under defined conditions. The development of coupled quantitative metagenomics and metatranscriptomics by Satinsky et al. (24) now allows the calculation of transcript abundances on a per-population level in the environment. The interpretation of these new environmental data sets will be greatly clarified and extended by the availability of baseline cellular transcript abundance data from environmentally relevant model organisms. The genome-wide inventories of transcript abundances produced here under a range of growth phases, nutrient regimes, and carbon substrates will better enable the physiological and metabolic interpretation of individual populations of OM43 methylotrophs *in situ*, as well as provide a knowledge base for predicting how the carbon-processing activities of these coastal methylotrophs may shift in response to various environmental conditions.

## MATERIALS AND METHODS

### Cell cultivation.

The basal growth medium consisted of Sargasso seawater that was subjected to TFF to remove cells, viruses, and HMW DOM (see reference 19). The nutrient regimes consisted of inorganic nutrients alone (30 µM phosphate and 400 µM ammonium [final concentrations]) or with an AMS1 vitamin mixture (see reference 27 for composition). To ensure the sterility of the seawater medium, which had been sterilized by TFF several months prior to these experiments, it (and nutrients and vitamins if added) was passed through a 0.22-µm-pore-size filter tower (polyethersulfone membrane; Falcon, Corning, NY). Subsamples of each medium type were then aliquoted into either 1-liter borosilicate glass bottles (regime I) or 500-ml polycarbonate bottles (regimes II to VI) and amended with either 50 µM methanol or HMW DOM from the North Subtropical Pacific (see below for HMW DOM description). The final concentrations of HMW DOM (measured in units of carbon assuming 0.4 mg of C/mg of DOM) depended on the availability of the purified product and varied from experiment to experiment as follows: regime I, 800 µM dissolved organic carbon (DOC), regime II, 2,200 µM DOC, regime III, 800 µM DOC, regime IV, no HMW DOC treatment, regime V, 400 µM DOC, regime VI, 560 µM DOC. The bottles were inoculated with an NB0046 starter culture to a final cell concentration between 500 and 2,000 ml^−1^, wrapped in aluminum foil, and placed in a dark incubator set at 22°C. Regime III included shaking at 60 rpm and bottle lids loosely screwed on to increase ventilation. Cell concentrations were monitored via flow cytometry (see Text S1 in the supplemental material). Generation time was calculated for each sampling point by dividing the time elapsed by the log_2_-fold change in the cell abundance ratio between the current and previous time points and then multiplying the result by 1.443 to get the growth rate. Cells were collected for RNA processing by peristaltic pumping onto 0.1-µm-pore-size filters (see Text S1). Assays comparing growth in small- versus large-volume chambers were conducted with 48-well microtiter plates with 1 ml of medium per well and in 10-ml polycarbonate test tubes containing 8 ml of medium.

### Methanol quantification.

An enzymatic method of determining the concentration of methanol in a seawater background by HPLC was developed. Subsamples of 10 to 20 ml were taken at various time points during the experiments and syringe filtered through a 0.22-µm Acrodisc filter (Pall; both the syringe and Acrodisc filters were triple rinsed with ultrapure water prior to filtering) into combusted glass vials. Filtered samples were stored at −20°C in the dark prior to quantification. Samples were thawed, and 1 ml was transferred into triplicate combusted glass HPLC vials. One hundred microliters of a freshly prepared alcohol oxidase solution (1.47 U/ml; Sigma Aldrich) was added to two out of three replicate vials and mixed before incubation at room temperature in the dark for 14 h. Controls receiving no enzyme were given 100 µl of ultrapure water prior to incubation to maintain consistent volumes. Methanol standards were prepared by adding HPLC-grade methanol to sterilized seawater in combusted glass volumetric flasks prior to the addition of alcohol oxidase. A 12.6 mM solution of 2,4-dinitrophenylhydrazine (2,4-DNPH; Sigma Aldrich) was prepared in a 1 N hydrochloric acid solution by heating the mixture in a combusted glass volumetric flask at 90°C for 8 h with periodic mixing. The alcohol oxidase enzyme activity was quenched by adding 40 µl of the 2,4-DNPH solution and incubating the mixture at room temperature for 1 h after mixing it. Chromatographic analysis was performed with an Agilent 1100 or 1200 series high-performance liquid chromatograph. Methanol standards and samples were injected (20 µl) and separated on a ZORBAX SB-C_18_ column (Agilent; 3.5 µm, 4.6 by 150 mm) by elution at 1 ml min^−1^ with a linear gradient (percent solvent A [ultrapure water], percent solvent B [HPLC-grade acetonitrile], time in minutes): 70, 30, and 0; 70, 30, and 2; 60, 40, and 4; 55, 45, and 16; 20, 80, and 18; 20, 80, and 22; 70, 30, and 24; and 70, 30, and 27. The derivatized formaldehyde peak was detected at 354 nm with a retention time of ca. 12 min. We corrected for any background formaldehyde present in each sample by subtracting the peak area (if detectable) of the control sample containing no alcohol oxidase from the average peak area of the replicates that received the alcohol oxidase enzyme. Methanol standards were used to derive molar concentrations of methanol from background-corrected peak areas. Using this approach, we could reliable quantify a range of methanol concentrations (100 nM to 50 µM) in a seawater background.

### HMW DOM.

The HMW fraction of DOM (>1 kDa) was concentrated by ultrafiltration from ~18,000 liters of filtered (0.2-µm-pore-size filter) surface seawater (15 m) pumped 2 km from the shore of the Island of Hawaii, HI, at the National Energy Laboratory Hawaii Authority in February 2013. The ultrafiltration system consisted of a stainless steel membrane housing and a high-pressure pump fitted with two GE/Osmonics 4- by 40-in. UF membranes (GE series) in parallel. Membranes were cleaned with 0.1 N NaOH and HCl and rinsed with 100 liters of seawater before use. Tubing and fittings were Kynar or polytetrafluoroethylene Teflon. Each day, approximately 1,600 liters of seawater was concentrated in a 200-liter high-density polyethylene barrel. At the end of sampling, the 200 liters was concentrated to ~20 liters, filter (0.2 µm) pumped into a 20-liter carboy, and stored at −20°C. The following day, this sample was combined with a new sample, concentrated to 20 liters, filtered, and stored. The sample did not freeze between collections but remained cold. The process was repeated until ~6,000 liters was concentrated, after which the sample was frozen and returned to the lab for further processing. The HMW DOM-concentrated seawater was then filtered through a 30-kDa ultrafiltration membrane to remove cell debris and viral particles, diafiltered to remove salts, and freeze-dried. A total of 10.6 g of freeze-dried HMW DOM was obtained that was 31% C (−21.6‰) and 2.8% N (6.9‰) with a C/N ratio of 12.9, representing 20% of the DOC in the original raw seawater.

The polysaccharide fraction of HMW DOM was concentrated by anion-exchange chromatography. A glass column (2.2-cm inside diameter) was slurry packed with 16 g of Bio-Rex 5 (Bio-Rad Corp.) resin (chloride form) and washed three times with 30 ml of 0.5 NaOH to convert the resin to hydroxide form. The column was then rinsed with water (80 ml) to a pH of ~6. HMW DOM (0.5 g) dissolved in 5 ml of water was applied to the column, and the carbohydrates were recovered by washing the column with 80 ml of water. Cations were removed by stirring with 1 g of Bio-Rex 50W-X8 resin for 1 h. The sample was filtered and freeze-dried three times to yield a fluffy white material. ^1^H NMR analysis of a concentrate sample was done to ensure that no methanol (<3 nmol/10 mmol of HMW DOC), formaldehyde, or formic acid was present in the sample.

### Internal standard synthesis.

Construction of the internal RNA standards was similar in approach to protocols described in references 23 and 52, with the exception that the DNA templates used for *in vitro* transcription (IVT) were generated directly from genomic templates (in this case, *Sulfolobus solfataricus*) via PCR amplification with T7 promoter incorporation. Regions of the *S. solfataricus* genome with little to no homology with the NB0046 genome were identified. Primers targeting these regions were synthesized and used for PCR amplification and T7 promoter incorporation (see Table S4 in the supplemental information for the primer sequences used). The RNA internal standards were generated from the template DNA amplicons via T7 RNA polymerase IVT with the MEGAscript High Yield Transcription kit (Ambion). The standards were quantified, pooled into groups, and added to the cell samples as shown in Fig. 3A. See Text S1 in the supplemental material for detailed information on standard construction, addition, and recovery.

### RNA processing and sequencing.

Total RNA was extracted via the mirVana microRNA isolation kit (Ambion) with modifications to increase reagent volumes (see Text S1 in the supplemental material), and residual DNA was removed with TURBO DNase (Ambion). Custom NB0046 antisense 16S and 23S rRNA probes were synthesized and used for subtractive hybridization of rRNA as described by Stewart et al. (53). rRNA subtracted samples were then prepared for sequencing with the ScriptSeq v2 RNA-Seq library preparation kit (Illumina, San Diego, CA, USA) and sequenced on the MiSeq platform (Illumina). For a detailed description of these procedures, see Text S1 in the supplemental material.

### Bioinformatics.

Sequencing statistics for each sample are summarized in Table S1 in the supplemental material. Sequences were processed with a local installation of the Galaxy bioinformatics platform (54). Several samples were sequenced over two MiSeq runs, and the FASTQ files for these samples were first concatenated with Galaxy’s “Concatenate Datasets” tool before any downstream processing. A custom Galaxy workflow was used to process the forward read from each data set (only the forward read was used to avoid counting errors and biases arising from poorly joined paired reads). Low-quality sequences and adapters were trimmed from the raw reads with Trimmomatic (trimmed to an aggregate score of ≥30). Trimmed reads passing quality thresholds were then mapped with Bowtie2 (parameters: end to end, sensitive, no trimming or skipping) (55) to three data sets simultaneously: the NB0046 genome (IMG genome 2562617047), the enterobacterial phage phiX174 genome (NCBI GI 9626372) to identify Illumina quality control sequences, and the *S. solfataricus* P2 genome (IMG genome ID 638154518) to identify internal standard reads. To obtain gene read counts, the Galaxy “Count Intervals” tool was used to tally ready counts from the mapped data on the basis of a genomic interval file containing coordinates of features of both NB0046 and *S. solfataricus* P2. Statistically significant differences in cellular transcript abundances among the four regimes’ late-exponential-phase time points were determined by ANOVA with the ANOVA() function in R (56) and correction of the resulting *P* values for multiple-hypothesis testing by the Benjamini-and-Hochberg method with the p.adjust() function. If a gene’s corrected ANOVA *P* value was <0.05, then a multiple pairwise *t* test of that gene was conducted with the pairwise.t.test() function in R to determine which treatments significantly differed, with *t* test *P* values of <0.05 and a minimum 2-fold change in transcript abundance considered significantly different.

### Accession number(s).

The sequences obtained in this study have been deposited in the NCBI sequence read archive under BioProject no. PRJNA330549.

## SUPPLEMENTAL MATERIAL

Text S1  Additional details of materials and methods, as well as a discussion of the transcriptional patterns in the four late-exponential-phase experiments for translational and iron-related genes. Download Text S1, DOCX file, 0.02 MB

Text S2  Supplemental data on internal standard sequences. Nucleotide sequences for the 14 internal standards are shown. Download Text S2, DOCX file, 0.02 MB

Figure S1  Strain NB0046 grown in seawater medium amended with vitamins and inorganic nutrients and in different-size cultivation chambers. Well, 1 ml of culture in a 2-ml well of a 48-well polycarbonate plate. Tube, 8 ml of culture in a 10-ml polycarbonate test tube. Download Figure S1, PDF file, 0.3 MB

Figure S2  Log-log plot of the recovery of internal standards in the sequence libraries versus the number of standards added for samples collected under all of the regimes used. Colors and symbols are described in Fig. 3. The regime and transcriptome sampled growth phase are indicated above the plot in the format regime number, growth phase, and replicate number. The fitted linear regression line is gray. Download Figure S2, PDF file, 0.04 MB

Table S1  Sample sequencing statistics. Table S1, XLSX file, 0.02 MB

Table S2  Transcript abundances (number of transcripts per 1,000 cells) for all of the genes in the NB0046 genome. Std., SD of biological triplicates; exp., exponential growth phase. Table S2, XLSX file, 0.5 MB

Table S3  Full names and IMG accession numbers of the genes shown on the Fig. 5 metabolic map. Table S3, XLSX file, 0.01 MB

Table S4  Fourteen primer pairs targeting the *S. solfataricus* internal standard sequences and incorporating a T7 promoter for the IVT (nonbold font) in the forward primer. Table S4, XLSX file, 0.01 MB
