# Supplementary material for: Quantitative Transcriptomics Reveals the Growth- and Nutrient-Dependent Response of a Streamlined Marine Methylotroph to Methanol and Naturally Occurring Dissolved Organic Matter
Source: mBio. 2016 Nov 22;7(6):e01279-16. doi: 10.1128/mBio.01279-16 (PMC5120137; doi:10.1128/mBio.01279-16)
Supplement: Text S1 — Additional details of materials and methods, as well as a discussion of the transcriptional patterns in the four late-exponential-phase experiments for translational and iron-related genes. Download [file mbo006163064s1.docx]

**Supplementary Materials and Methods**

**Internal standard synthesis.** Construction of the internal RNA standards was similar in approach to protocols described in Gifford *et al.,* (2011) and Satinsky *et al.,* (2013), with the exception that the DNA templates for the *in vitro* transcription (IVT) are generated directly from genomic templates via a PCR amplification with T7 promoter incorporation. *Sulfolobus solfataricus* genomic DNA (35092D-5) was obtained from the American Type Culture Collection (ATCC®) and the corresponding genome sequence (*Sulfolobus solfataricus* P2, IMG TaxonID: 638154518) was downloaded from the Integrated Microbial Genomes (IMG) database. Potential internal standard sequences were selected from within the *S. solfataricus* genome, and any significant homology between those *S. solfataricus* template sequences and the NB0046 genome were identified using CLC genomic’s *Find Binding sites and create fragments* tool (8 nt minimum identity, 8 nt required a the 3’ end). Any *S. solfataricus* template sequence with ≥ 15 nt identity to the NB0046 genome was not considered. The selected internal standard sequences passing these criteria are provided in the file: *Supplemental Data Internal Standard sequences.docx*.

PCR primers targeting the selected *S. solfataricus* sequences were designed using the CLC genomics workbench *primers and probes* toolbox with the addition of the following T7 promoter sequence to the 5’ end of the forward primer:

5’ *–* GCCAGTGAATTGTAATACGACTCACTATAGGG - ForwardPrimer - 3’

The full list of primer sequences is provided in Table S4. These primers were then used to PCR amplify the DNA templates from the *S. solfataricus* genomic DNA in 50 µl reactions consisting of 2.5 µM each forward and reverse primer, 1 µl of Herculase® II Fusion polymerase (Stratagene 600677), 10 µl Herculase 5X buffer, 34 µl water, 2 µl *S. solfataricus* genomic DNA (~100ng), and 0.5 µl dNTP (100mM). PCR reactions were conducted in a thermocycler with the following conditions: initial denaturation at 95°C for 2 min followed by 35 cycles of denaturation at 95°C for 20 s, annealing at 55°C for for 20 s, extension at 72°C for 1 mins, and then a final annealing for 3 min. After amplification, the PCR products were purified using the QIAquick PCR purification kit (Qiagen) and run on an Agilent (company) High Sensitivity DNA chip to verify a single fragment of the target length.

The RNA internal standards were generated from the template DNA amplicons via T7 RNA polymerase *in vitro* transcription (IVT) using the MEGAscript High Yield Transcription kit (Ambion) following the manufacturer’s instructions. The IVT reaction was incubated at 37°C for 2.5 hours, after which the DNA template was degraded by adding 1 µl DNase to the reaction and incubating for 30 mins at 37°C. The IVT products were purified using the MEGAclear kit (Ambion). Verification that the synthesized RNA standards were single fragments of the target length was determined by Agilent’s Prokaryote Total RNA Pico chip. The concentrations of the RNA standards were determined by RiboGreen (Invitrogen), and then diluted and pooled into one of the four internal standard groups (Fig. 3A). Standards were added at a 1:100 standard RNA to sample mRNA ratio, assuming 1 µg total RNA in the samples of which 10% would be mRNA.

**Generation and recovery of internal RNA standards.** The majority of standards were recovered in the expected proportions, though three standards had notable deviations. Standard 5 (group A) was an average of six fold higher than expected (Fig. 3B), and given the rest of the standards in its group are represented in the correct proportions, the high abundance of standard 5 is likely the result of an error during the original quantification or due to a potential bias during sequencing. Standard 4 (group B), conversely, was recovered on average of 30% less than expected based on recovery of the other standards. Standard 2 (group C) was added to the samples at the lowest concentration (378 000 RNAs per sample), and was not detected in several of the samples, which was fitting given many of these samples had a lower detection limit 100,000 transcripts per sample based on internal standard recovery. Given these potential biases, standards 5, 4 and 2 were not used in any downstream calculations. The remaining 11 standards were used to convert transcript reads per library to transcripts per cell by taking the average conversion factor (standard molecules added / standard reads sequenced) of all 11 standards within a sample.

**Monitoring Cell growth.** Cell concentrations were monitored over the course of the experiment by removing 50 µl aliquots from the bottles, mixing with 140ul of sterile seawater medium and 10 ul 200X SYBR green I stain, incubating for at least 45 mins in the dark at RT, and then quantified using a Guava 8HT flowcytometer.

**Cell collection.** Collection of cells for RNA processing was done by pumping the culture through a sterile serological pipet and tygon tubing using a peristaltic pump and through a 47mm, 0.1 µm pore size filter (Durapore, Millipore) in a swinex filter holder. The weight of the bottles before and after filtration was used to measure the volume filtered. Upon completion of filtering, the filter was removed and placed in a cryovial and submerged in liquid nitrogen. After several minutes, the cryovials were transferred to a -80°C freezer. Total time from the start of collection to flash freezing was 8 to 10 min.

**RNA processing, library preparation, and Sequencing.** Total RNA was extracted via the mirVana miRNA Isolation kit (Ambion). After removing the filters from -80°C, 1.1 ml mirVana lysis buffer was immediately added to the tube and vigorously vortexed for 1 min. Each of the four internal standard sets was then added to the sample in independent, 100 µl aliquots (for a total of 400 µl standard volume). 150 µl miRNA homogenate additive was added and the tubes were incubated on ice for 10 min with occasional mixing, after which the filter was removed from the tube and discarded. Each sample was then split into two aliquots of 825µl and the rest of the extraction proceeded according to the manufacturer’s protocol, eluting in RNAse free water. Residual DNA was digested by incubating with 3 µl TURBO DNAse (Ambion) and 10 µl DNAse buffer for 20 min at 37 °C, followed by the addition of 3 more µl of Turbo DNase and another 20 min incubation at 37 °C. The reaction was terminated using 20 µl TurboDNAse inactivation reagent.

Antisense 16S and 23S rRNA probes for subtractive hybridization were synthesized as described previously by Stewart *et al.* (2010). To generate templates for antisense-probe synthesis, DNA was extracted from a mid-exponential NB0046 culture grown on 300 µM methanol and used as a template in PCR reactions with universal primers targeting the 16S and 23S ribosomal RNA subunits and containing a T7 promoter (see Stewart *et al.*, 2010 for primer sequences). PCR reactions consisted of 1 µl Herculase® II Fusion polymerase, 10 µM forward and reverse primer, 1 mM dNTP, 1X Herculase® buffer, 5 µl genomic DNA template (~100 ng), and 31 µl water. Five replicate reactions were setup for each rRNA subunit. PCR reaction conditions consisted of 95°C for 2 min, followed by 35 cycles of denaturing at 95°C for 20 s, annealing at 55°C for 20 s, and elongation at 72°C for 2 min, and a final extension of 72°C for 3 min. After amplification, replicate reactions for a given subunit were pooled and purified using the QIAquick PCR purification kit (Qiagen). Biotinylated antisense rRNA probes were generated from the PCR amplicons by *in vitro* transcription (IVT) with T7 RNA polymerase using the MEGAscript High Yield Transcription kit (Ambion) in separate in 20 ml reactions as described by Stewart *et al.* (2010), with a DNA template input of 500 ng 16S or 23S PCR product. Reactions were run at 37°C for 4 h, then DNAse digested with TURBO DNAse (Ambion) for 30 min at 37 °C.

Total RNA from the transcriptome samples was then depleted using the synthesized anti-sense RNA probes and the subtractive hybridization protocol described by Stewart *et al.* (2010). The antisense 16S and 23S rRNA probes were each added to the hybridization reaction in a 2:1 ratio of rRNA probe to total RNA sample. After subtraction, the RNA was purified using the RNeasy MinElute kit (Qiagen), and the efficacy of subtraction determined by a 2100 Bioanalyzer (Agilent, Santa Clara, CA, USA).

rRNA substracted samples were then reverse transcribed to cDNA and prepared for sequencing via the ScriptSeq™ v2 RNA-Seq Library Preparation Kit (Illumina, SanDiego, CA,USA) following the manufacturer’s protocols. Individual samples were barcoded by replacing the reverse PCR primer included in the ScriptSeq™ kit with the ScriptSeq Index PCR Primers (Illumina). Samples were sequenced using the MiSeq platform and V2 kit reagents (Illumina). The nutrient deplete samples (ExpI and UVI) were prepared with the paired 100x100 MiSeq reagent kit, while all other samples were prepared with the paired 250x250 MiSeq reagent kit.

**Supplemental Results and Discussion**

Differential expression of translational and iron related genes:

**Chaperones***.* Chaperone protein transcription was relatively low under the nutrient deplete regime, but when inorganic nutrients were added to the medium, chaperone transcripts were significantly enriched, including increases in the *dnaK-dnaJ-grpE* complex, as well as the secondary *groE* chaperone complex (*groEL*, *groES*; Table 1). These chaperones are required for proper folding of nascent proteins and correct protein configurations under environmental stress. Previous studies have shown that when nitrogen stress is relieved in *E.coli* (38) or Xylella fastidiosa (39) the cells significantly increase the relative abundance of chaperones and ribosomal proteins. This suggests chaperone expression might be a part of the NB0046 nitrogen starvation response, such that when nitrogen stress is relieved and new protein production is less constrained, chaperones are upregulated to ensure correct folding of new proteins.

**Iron.** The addition of inorganic nitrogen and phosphorus to the cultures also led to changes in iron related transcript abundances, primarily those involved in incorporating Fe-S centers into newly synthesized proteins via the iron-sulfur cluster (ISC) system. This included transcripts of *IscA*, which acts as a scaffold to accept sulfur and iron, which are then passed on to the molecular chaperone *hscA* (also significantly enriched) and *hscB* (not significantly enriched) (Table 1). Two *tonB* receptor transcripts were also significantly enriched upon inorganic nutrient addition. One of these *tonB*s (NB46_01062) is annotated as a putative siderophore transporter and its transcription increased by 40 fold in the nutrient amended-methanol regimes. The other *tonB* gene (NB46_00103) increased 4 fold in the nutrient amended regime and it neighbors a bacterioferritin that had the opposite transcriptional pattern. This suggests that relief from nutrient stress may have resulted in increased uptake of iron, as well as moving iron from storage pools into newly synthesized proteins.
