# Supplementary material for: Quantitative Transcriptomics Reveals the Growth- and Nutrient-Dependent Response of a Streamlined Marine Methylotroph to Methanol and Naturally Occurring Dissolved Organic Matter
Source: mBio. 2016 Nov 22;7(6):e01279-16. doi: 10.1128/mBio.01279-16 (PMC5120137; doi:10.1128/mBio.01279-16)
Supplement: Text S2 — Supplemental data on internal standard sequences. Nucleotide sequences for the 14 internal standards are shown. Download [file mbo006163064s2.docx]

>s1_arsB_v1_1068_frag

CCTTGGGCTTCTATGTTTTTTGGTGGTATCCTTATGGTAATTCTAGGTATAATATCTCCA

GAAGAAGCTTTACAATCAATAAATTTAGATGTAATATTATTTCTCATTACCCTTTTTACA

TTTGCATCAGCGTTAGAGGTTTCTGGATTTTTGAAATTCCTTGCATATAAGATTATAGAA

AAATTCAAGGAACCTAGGAAAGTTCTCTTCTATATTCTTTTATATTCTGGTCTATTATCA

AATTTAGTTACCAACGATGGAGTATCAGCAAGCTGGACTCCAGTCATCTTAGAATTAAGC

AGGATGATAGGCGTTTCTGAGGTTCCTTTTCTTTATGCATTAGCTGTTGGTGTTACTATT

GGGAGCGTTATAATGCCTACTGGCAATCCTCAAAATTTACTCATAGCTTTAGAATCTGGA

ATAAAAAACCCTTTCATTACATTTACAATATATTTGACCTTACCCTCAATAATTAGCTTA

ATAATTGCTTATTTTATACTCTTTCGTCTATTCAGAAAATCCTTGTCTTTACCAAGTGGA

ATTAATATAAAAAAAGAAGAAGAGGAAAAGGTTGATTTCGATAGAAGACTTGGATATCTG

ACATTAACCTTATTAGTAGTTACCATAATATTATTTTTTTCCTTAAGTTTCTTTAAAATA

GATATTTTACTGGGTTCTTTAGTTACTTCATCTATCTTACTGCTTTTAACGGAAAAGAGG

AGGGATATTGTAAGAAGAATGGACTGGCCAACTATACTATTTTTTATCGGATTGTTCATA

TTTACTGATGGAGTATTAAAATCTGGGATTATACAGTATTTATCTAATTTTCTTCCCCCT

CCAGATAGTGTGGCTAGTATAATGATTGTAAGTATTTTACTGAGCCAAGTATTAAGTAAT

GTACCATTGGTTGCAATATACATACCGATCATGATCTCTCATAGTGGTATTACAGTGGTG

GATTGGCTAGCGTTAGCTGCGGGTAGTACTATAGCGGGTAACTTCACCATATTAGGCGCA

GCAAGTAACGTAATAATTTCTGAAGCTTCTGAGAGCAGAGGTGGAAAA

>s2_huyA_v1_1077_Frag 638162896 N-methylhydantoinase A related protein GGAGAAAGGAAAAACGAAGTAGATACGCTAATGATAGGAACTACTCACGGTCTGAACGCC

TTACACCAGGGTAAAGGCTTAAATAGAGTAGCGACCATTAGAATTGGCTTACCTGCAGGA

GAGGGAGTTCCTCCAGTATTTGACTGGCCAGAGCAGTTATCAAACTTTGTCACCTATAGA

TATATGGTAAGAGGAGGCCATGAATATACCGGGGAAGAAATAGTGGAGTTAGATGAGGGC

AAAATAAAGGAGATTGCTGAAGCCATAAATGGTAAAGTTGATGCCATAGCTATTAGTTCA

ATATTTTCAGTTGTAAATTCGTCACATGAGATTAGAGCGAGGGAGATTTTAAGAGAGAAA

GGAATTAATGTGCCTATAGTACTTTCTCACGAAATTGGTGGAATAGGACTGTTAGAGAGG

GAGAACTCAGCGATCCTAAATGCGTTAATACTTAAAATCTTCGATAACTTAATAAGCAAA

ATCAAACAGTTACTTTCTTCTTTAGGTATAGAAGATGTGAGACTATTCTTTGCACAGAAT

GATGGGACTGTGGCCTCTGAAGATTTCATCAAAAGCTATCCAATATTCACTGTAGCTGGA

CCAGTTTCAAATAGTATTAGAGGAGCGCATTTACTGACTGGGATAAAAGATGCAATAGTA

ATGGATGTAGGAGGGACTACAACAAATGTGGGTGTTCTCCATGAGGGATATCCTAGAGAA

TCCTCATCTGTAGTAGAAATAGCCAAAATAAGGACTAATTTTAGAATGCCCGACATTTAT

ACGATGGCATTGGGAGGAGGCACCATAGTTAATAAGGAGAAAATAGGACCAGAGAGTGTG

GGTTACGCACTGATAAATAAGGGAATATCATGGGGAGGTGATACTTTAACCGCAACAGAT

GTAGCTATGATAGTGAAAGGAATAACAATAGATGGTACAAATCCGAAGCTAGTAAACAAC

AAATTCCCTATGGAGTACTTATTTAGCGCATACACTAAAATGGTGGAAATGTGGGAAGAC

GCCATAGACTTAATGAAAACTTCAAAGGATGACGTAACGGTAATTGTTGTGGGTGGG

>s3_pdhD_v1_1000_Frag

AGAAAGGGCAAGAAGGTACTAGTTGCCGAAAAAGAGAAATTTGGTGGAGTTTGTGTAAAC

TTCGGCTGTGTACCAAGTATTTTTCTTTTTGATGCAACCTTTTTATTAAATAGATTTAAA

GAAATTGTGTATTATATAGGCTTAGATGGTGAAATTGAATATAAAGATCTCCTTTTCAGT

AAAAGAAACGAAATTATAGACTACTTATCAAACGCCGGAAGAAAGCTAATTGAAGATTCG

GGCGGTGAAACTGAGTTAGGTGAAGTTGAAATAATTTCTCCTAGTACGGTAAAGGTAAAT

GGGAGAATTGTGGAGTTCGATAATTTAATCATAGCTACTGGTTCTAAACCGATGGTACCA

AGTATTAATGGCATTGAAAATACTTTAAGTGAAGATGATGCGGTTAATTTGAATTCAGTA

CCTTCTTCAATGGTTATTATTGGCGGAGGCTATGCAGGAGTTGAGATAGCTCAAATGTAT

TCCAGATTAGGGTCACAAGTTACCTTATTGTCCAGAAGTAAAATTTTACCAACATTTCCA

GAGGATGCTAGAAGTATTATAAAGGATTCCTTGGAATTCGATGGAGTAAATATAGAGGAA

AACATTAGAATAGTGAAGATTCATGATGGAAAAGTGATTACGGAAAAGGGTGAGGTAGAA

GGAAACGTAATAGTGTATGCCACAGGGAGAAGACCGCAATTGCCTAAGGGTATTGAAATA

CTTGGATTGAGTATTAATGAATGTGGGATAGTAGTTGATAAGTATAGAAGAGTAAAGAAC

AATGTATACGCAATAGGTGATGTAATAGACAAAGAGAGAAAAACTGCACATTCAGCAATT

TTAGATGCAGTAATTGCATCACTACATATCCTTAAGGACGCAACATTTCTTCCCCTAATA

GATAATCTTAAGATACCCCAAGTGTTATATACTGATCCTCAAGTTGGTATCGTAGGTAAC

GATAAAGAGGCTAAGGAGTTTTCTGTTTTTCCGTTTGCTG

>s4_mcrB_v1_1028_Frag 638162545 Restriction endonuclease related protein

GAACAAAAGAGCTAGGCATTATTGGATTCGGAGTAGTAACGGATATCACAAAGGACGCGA

TGAGGAATTTTAAAGGATGGAAGGAAGGTGACAAAGTCTGGATAGTAAGATTTAGAATAA

AAATATTTTGGCTTCACGAAAGCATAAGAAAAAACTATAATGACAGTAATAAATGGGTGG

GAGAAGATTATAAGTTAGAAGGGGTTAATAATCAATCAAACCAATGCTATACAGAAGACA

ATAATGATGCAGTTAAAGCTTTTCAACAATTTATTCTATCGAAGAAAGAGGAAATTAGGT

CTACTTTAGAATTTTATTCTAAGTTTCGTTTCAGTACTCAGAAAGAATACCTATCAGAGG

AAGTAGTTTGTAATAAAAACGCACAAATAGATCTTAATGGTTTCTATATTCCTCAAGATT

CAGTTAACATTATACTGAAAGCTCTACAAACTACTAATGTACTCCTAGCTGGCCCACCTG

GTACTGGAAAAACTAGCTTAGCTATAAGGACTGTGAGAGCTTTAACTGGTAATAATGATA

ACTGTTATGAAGTAGCTACTGCCAATTCACTTTGGTTTAGAAGGAATTTGATAGGAGGAG

AAAGTATAAGAGAGGGATCTGTAATGTGGAAGAGCGGGCTTTTCATTGAAGCTTACGTAA

ATGCTGCAAGAATAAAGGATGGGAACTATTACGTCGTAATTGATGAATTGAATAGGGCTG

ACGTTGATAAGGCATTTGGAGAATTAATGACAATATTTTCTACTCCATATCCTAGCGAAT

GGTCAATTCCCAATGCTCTAATAGAGGAAATTAAGAGTTATGGAGATAATATAGATAATG

TAACCAAGAGTTTCTTAGAAATATATGAGAAGTTAAAGAGGGAGAGTAGAGAGAATGAAC

CATTAAAAAGAATACGAATTATTGCTACAATAAACTTAGTCGATGCTAGAAATCTGTTTT

ATGTTGGAGATGCGCTGGCAAGAAGGTTCGTGATAATTTACTTTGATTATCCGAAAGAAA

CCGAAGAT

>s5_hdrB_v1_979_Frag

AAGCCCTACAATAACCCAGTAGAAGCCCAAACACTAAACGGATCTCCAAAGAAAATACCC

ACAACAAAATTATGGCATCATAAGAGTTGTGGACAGTGTGGCCACATACCCGGTTATCCA

ACCTCTGTTTTCTGGGTAATGAATAAGTTAGAAATAGATTACCTAGATGAACCGCATCAA

ACATCGTGTACTGGATGGAATTATCACGCGTCTGGTGCCTCCAACCCCGTAGCCTTGGCA

GGAGTATATGTAAGGAACATGTGGAGAGCTTATGAAACGGGTTACTTCCCATTAATACAT

TGCGGAACATCATTTGGTCATTATAAAGAAGTTAGAAACATGATAATATTACACAAAGAG

ATAAGAGACAAACTTAGACCAATCATGAGAAAACTGGATATGGACATTGTAATACCAGAA

GAGGTAGTTCATTATTCTGAATGGTTATATGTAATGAGCAAGAAAGCTGCACAGCAGAAG

AAATACAATCTAGATAATATTAAGGCAGCTGTACATACTCCTTGTCATGTTTATAAGTTA

GTTCCAGAGGATACTGTTTACGATCCCGAAGTATTCCAAGGTAGAAGACCAGCAGCCCCA

TCTGGAACTGTACAGAATTTCGGCGCTAAACTAGTGGATTACTCAACCTGGTGGGATTGC

TGTGGATTCGGATTTAGACATATCCTAACAGAGAGGGAATTCAGTAGAAGTTTCGCACTA

TTTAAGAAGGTTATACCAGCAGTTGAGGAAGGGAATGCTGATATCTTTGTAACCTCAGAT

ACTGGGTGTGTTACTACTTTAGATAAGAGTCAGTGGGCTGGAAAGGCTCATGGTTTCAAT

TATAACTTACCAGTATTAGCTGATGCGCAATTTGCAGCTTTAGCAATGGGCGCTGATCCA

TATATAATTGCTCAAATTCACTGGCACGCGACAGATGTAGAAGGTTTCTTAAGAAAGATA

GGTGTTCCGGTTGATGATT

>s6_hyp1378_v1_964_Frag

GCGAAACACATACTCAACTTCTAAAGTGGAGGGCGGAAGTAGTAGAAGGAGGGAGTATAT

CACCTAAGAACCTTGAGACTCCATTAAATAGCATGAATAGCTCTACTGAAGAAAACGTTG

AGAGTATAGTAATCTTAAAGAGGAGAATAGATGAGTTCAATGAGATTGTGAAGGAAGAGG

AGTCATTTTTAAAGAGTCCTCCTGCAAGCCAGATAGAGTACGTCTTTCCAGTTAGAGAAC

TTGAGAATTTTAACGTATCCCAGTTATCCACGGACAAAGCATCACTTTATAGACTAATCG

AGGAGTATAGGAGAAAAGACGAATTCTTAAGAGCTTTAGCAGACTTAAAAGCTCCACTAG

GTTCAGTAGGGCAGACAATGGCTAATATCGATTACCAGAAAATTAACGTTCCTATACCCA

TAACTACCGAGGCTAAAGAATTCATTTCTAAGTACAATTCTGGCTTAATAAAGCAGAATG

CAGTAGTATCCCCCGATATAGCTAGCGTATTGATGGTTACTGCATCAATGCAGGATAATA

TAAACGTTAATGATTTTCCTAATCCAGAAACCCTTAAACAAGCGTTAGAGAGTAAGACTC

GCAATCCAGACGCTAAAATGGGCTATATCAATTCAAAAAGATTTAGTATATCCACATATC

ACATAATAAATGGTGTTTACATATGGAGAATATCTAAAAATACCCCTCCTATTCTAAGGG

ATTTAACGTACCTCATAGAGGATTATGAGGCTGTTAAGAACGAAAACTTTGGAGAGTTAA

TTTACCATCATACATTGTTTTATAATGATCCTCAGACCTTTGAAGCATTAACGGGAGAAC

AAATAGCCAATTTATCCCCTAATCAAGCAGCTTGGAGGATAATAGAATTTTGGGCGAACT

ATAATCCAGAAATAGAATATATTAATATTTGGGGAATTATAGAATTAGCCGGCGTATATA

GTGA

>s7_hyp1273_v1_991_Frag

ACACTACTAAGCTTCCAGACATATTATTCTATAACGAATGGAAGCTATCCTAATGTTACA

ATAGGTGTTAGATACTTAAGTCAAGGTTCGGCTAATCCCGGTGCTGGTTTCACTGATTCC

TATACAGATGAAATAGGAAACGCTTTATTCACTCCGTCCTCTTTAACAGTACCAGGTTCG

GGATACCCAGTACCTTTCATTTATACTTACAAGATAGTTAATATAACTCCACATGCTGTA

GTATCAGTACCTTCAAATGCATTGTGGTGGAATCCAACTACGCAGCAAATAACTAAGGTA

TCTCCTAATACTACTGCGCAAATGGCTGTAATATATAACTTAGCCCCACTGTTCAATAAC

GATAAATGGGCTGATGGTCAAAATATAACTCTTGCTGACATAATATATGAATATATTGTT

GCATCCGAGATGTCCTTAAACTCTAGTAATCCAATTTATGACTCAACTGCATCATCAGTA

TATGCTCCAGCACTACAGACTATTAAAGGATTTAAGATAATTAACTCCAGTGCTATAGAA

ATATGGGGCAATGATTGGTTCTTCGATCCTACTGAGGCTGTAGTTAGCTTATTTGGATCT

TTCAATCCACTAGGTTACGCGTTAGCTGGCGGAGGTTATTTCCCATGGCAAATGTATGTT

GGTATGAAAGATGTTGTTGCACAAGGTAAAGCTGCGTGGTCTGAGGGAGCAGCTCAGTCC

AAAGGAATTGATTGGCTGAATCTAGTTAGTCCAACTGATATTGGATATATAACTTCAGCG

TTACAGAATGCCTCAGCTACTGGATATATACCTAAGAGTTTACAGATAGTAGAGAACTTA

AGCGGTATAACTCTAGTAACTCCACAAGAAGCAAAAGCTGGATATGAAGCGGCAATTAAC

TTCATGAAGACCTATGGAAATGGATTAATAGGTGATGGTCCATATATCTTGGTAGCGTGG

AATCCGAGTGCGTCTCCACCCTACGCAAAAC

>s8_tri3_v1_1000_Frag

GGCTTATGTTGGTGAGGAGAATTTCAGAAGGGGGGTTGTCAATTACTTAAATTCTTTCAA

ATTCGGAAATGCAGAGGGTAAGGATTTGTGGAATTCTATTTCTAACGCAGCTGGGCAGAG

TATTGGAGAAATTATGGCTGATTGGATTACAAAGCCTGGTTACCCTGTAATTTTTGTCAA

CGCATACGGTAATTCTATCAGGTTTTCTCAAAAAAGATTTACACTTCTTGATAGCGGTTT

AAATGAGGTTTACAAGGTTCCAATTACATATGAGATTAATGATAAATTTGGCACTCTTCT

TCTGGACAAGGAATCAGCTGAAATAAGGTTAGATGAAGGTTTGAAGAGTATTAAGGTTAA

TATAAATAGGACTGGATTTTATAGGGTCCTTTATGATTCTCTTAATCTAGCTTTCTCATC

AAAGCTTAATGCTTATGAAGAGTTGGGATTGGTTAACGACTATTGGAATTTCCTATTGGC

TGATCTAATAGATGCGAAGACGTACTTTGGCGTAATTGGTAGGTTTGTATATACTTCTAA

CTCTTTTGTATCAAGAGAGATAACCTCTCAACTCTTAACATTATATTATCTATTTAAGAA

AAATTACGGGAAAGATTTCCTAGTTAATCAAGTTAAGATATTTAGAAAGGCTAATGACGA

CCTAGGCAAATTAGCGTATTCAACTGTTATCAGTGCCTTAGCTAGAATGGATGAAGAGTT

TGCATTAGGATTATCAACTTTATTTGATCAATATGAAAATATAGACAGTAATATTAAAGA

AGCTGTTGCAATAGCTTACGCAGTAACTAATAACGACTTCAATACTCTTCTAGAAAAGTA

CAAGAGGTATACAATAGATGAGGAGAAGAATAGAATATTAAGTGCAATTTCATCACTTCG

TGATCCATCAATTGTAGTCAAGGTTTTCTCACTAATATTTGAAAGGAATATAAAGGCTCA

AGATACTAGATTCGTTATATCTTCACTGCTACACAATCCT

>s10_hyp1806_v1_969_Frag

GGAGGGATTAGGGTTTTAGGACCAAACACCTTTGGAATTATTACACCGGAATTTAATGCG

ACCTTTACTTACACCGACGTAAAGAGAGGAAATGTAGGTTTAGTGGTTCAAAGCGGTGGG

CTAGGAGTTTATATGTTGAACTGGGCTCAGAAGTATCGGATAGGGATAAGCTATATGGTG

AGTTTAGGCAATCAAGCTGATGTGAAGGAATATGAAGTCATCAATTACCTATCAAAGGAC

GCTGAGACTAGAGCAATATTCGTTTATTTAGAAGGGGTATCAGATGGAAATGCTTTCCTT

GAAGAACTACCCGAGGCTACTAGAAGAAAACCTGTGGTCTTTTTGAAAGGAGGAGTCAGT

AGTAGCGGAGCTTCGGCTGCTAAAACACATACTGGCAGTTTAGCTGGATCATTTGAGGTG

TTTAAAGCAGCTGTTAATACAATAGGTGGGATCTTAGTGGATAACCTTCACGACATGTTA

AACTTGGCTAAGATATTAATGTACTCTGAACCGATAAGTGAGGAACTCTTAGTTATAACT

AACTCTGGTGGACATGGTGTACTAGTTTCTGATGAGATAGACAAAAATGGGCTTAGACTG

GTGGAAATCCCAGAATGGATGAAGAAAGAACTAACTAAAATACTCCCACCTACATCCCTT

CCTAAAAATCCCCTTGATCTAACTGGAGACGCCGATAGGGAGAGATATCACAATGCGTTG

AAAATCGTTAGCAGTCTAGATTGTACTAAATTGGTGATAGTACAGTCCTTACCCATGGTA

AGTTGTAGTGACGTTGCTAGGGTTATATCCAATTTCAAAGGGAAAGGAGTGATTGGCGTA

ACCATGGGCTTAGATGAGGATATGGCATTAAAAATATTGGAAACGACCGGAATTCCAGGA

TATACTTTTCCAGAGGATGCTGTAAAAGCGATTAAATATTACACTTTTAGACCAACACCC

AGAAAGAAA

>s11_tynA_v1_996_Frag 638162924 Amine oxidase (copper-containing) (tynA)

GCCGCTTATCCACCTATTACGTTAGATGAATTTGGAGAATGTGAAAAAGCAGTTCGAAAT

GACAAAAGAGTACAAGAGGCTTTGACTAAAAGGGGAATCTTAATAAACGATTTGAACTTG

CTAATGGTTGACTGTTGGGCTCCCGGTCATGTTGACGAGGAGTTGCGAGGTAGAAGGGTA

GCAATAGGGTATATGTGGGTAAAAAAAGATATAGAGGACAATGGATATGGTAGGCCAGTT

CATGGGCTTATGCCTTGGGTTGATCTAGATAAAATGGAGGTTATCAGAATAGACGATCAT

GGAACTTCACCTCTCCCATTACAAGATGCTAATTATACGCCTGAAAAGCTAGGTAAGATA

TTTGGCGACGATCTGAAGCCTATTGAGATAAGACAACCATTATCCTCAAGTATTAAAATT

AATGGTTGGGAGATAAGTTGGTATAGATGGAGATTAAGAATTGGTTATACACCTAGAGAG

GGTTTAGTTATTTATGATGTTAGATATATTGATGAAAATAATCGAGAAAGAATGATATTA

TATAGGGCTTCTGTAGTGGATTTAATGGTACCTTACGGAGATCCCTCTCCATTCCATAAT

AAAAAAATGGTGTTGGATGCAGGTGATTATGGGTTGGGAAACTTTATTGTGCCATTATCA

CACGGAAACTATGATCTATATAACTGTGATTGTTTTGGAGAAGTTATATATCATTTAGAT

GTGACAAGGGTTAGTTCTAATGGGACTCCTATTAAAATAAAGAAAGCTATTTGTGTTCAT

GAAGAGGACTTTGGAGTCTTATGGAGGCATACCGATTTAAGAAGTGGTAAGTCCGAGGTA

AGAAGAAACAGAAGGCTTGTTGTCTCATTTTGGGCGACGTTAGCTAATTACGATTATGGA

TTCTTCTGGTATTTTTACCAGGATGGCAGTATCGAATTTCTAGTAAAACTAACTGGAATA

ATAAACGATGATAGTATAAGCGAAAAAGACCCAACA

>s12_hyp1079_v1_991_Frag 638162307 Hypothetical protein

TGGGGAACTACAGAACAATTAATATTTGCTCAAGTAGGAGAAGGCTTAATACCTCTTACC

TTTAGCGTTTTTAATCCCACGACGGAACCCATGTTAAACGTAACCTTAAACGTAGTCTTA

CCCAAGGGAATATTTACCCAAAGTGGTAGTAGAGATTTAACAGTTACAATTCCAGCCTTA

CCTGCTGGAGAACCAATATTCGTATCTTCAATAGTAAATGTAAGTAACCTTGTTAAACCG

GGAGTGTATAGTCTTAATTATTCCTTTTCATTTACGAACTTTCTAGAATATTTCTACAAG

CAAGAATCAAATAAAAGTATAAATATAACAATATATCCTCAAGCCAAGATTAACATTTTT

TCCGATCCCATCCAGTCAACTCCAGATAATATAACTACACTCGTAATAGGAATTTCAACT

AACGTTAGTAGCTTTATAATTAGTGTAAAGCCAGAATTGCCCTCAAGCTTCCTACCAATA

TCCTCAAACTTTACGTCTACTTCTCTATCACCGGGAGAAAAAGTAATATATGCTTTCAAG

ATTTACATACCTCAAGGGGTAATACCCTCAATATACCCTATACCAATAGAGGTTAATTAC

AGTGCATTGGACCAAAAGCTAGCAGTAGTTTATCTAACATATGCAAATATCTACTATAAT

GAAACTCCTAGGGTAGTTGAGGCAATTTGGAATACTACGATAACTCCATTTCCAGGGATA

GGGACAATCCCCTTAACATTGGTTATCTATAATCCATTACCAATACCAATAACTGGTGTT

AATATTACATACAAATTTCCTAATGGTGTATTTCCTTTACAGCCATTCATTTTTTTACCT

GGAATTCCTCAGTATTCTTCTGTACCAATAACTATCCCAGTGGAAATAACGCCAAATGCC

TCAGTAGGCGTACTCAATTTCACTTATAAGATTTCTTACAATCAAGATAAAACTGTTGAT

GGCGTAAATAATATTTATGTTTTGCCCCCTG

>s13_therm_v1_613_Frag 638163115 Thermopsin precursor, probable

GGAGTATATGTTAGTTTCGGTTATGTTATTCTTCAAAACGGAAATATAAGTCCACCTAAC

CCAATATTTTACGATACGGTCTTCATTCCAATTCAAAATTTATCATTTGCTTCAATTATA

ATAGCTAATCAAACCACCCCCAGCGCGAATTTTGGTATTGTTACATATCTGGGAAATTAT

TTAGATGCTGAGTTAGTATGGGGAGGATTTGGGAATGGTGAAAGCACAACTTTCTTAAAC

ATGTCTTCTTACTTAGCATTACTCTATATGAAAAGTGGCGAATGGGTTCCATTTTCACAA

GTATACAATTACGGAAGTGATACCGCAGAATCCACTAATAATTTGCAAGTTTTGATAGGT

AAAAACGGTGATGCTTACGTTACAATAGGCAGACAGAACCCTGGTCTATTGACTACAAAA

TTTAACCCTTCATATCCAAGTTTCCTATACTTAAACATTAGTAGCAAAATACCATTTCTA

CTAAATAAAAGCCTTTCACATGCATTCTCCGGCTACGTTACCACCCAAATTAAATTAGGA

TTCTTTAAGAACTATTCAATTAACTCATCGTCATTTGCAGTGCTTAATGGAAACTATCCC

AGCCTAATAGAAC

>s14_virB_v1_301_Frag 638161336 Type II secretion system protein, virB homolog (gspE-1)

TAGCTGACCCTTATGTTGAAGAAATAGAATGTAAAGGATTCTCATACCCTGTCACTGTAG

TTCATAGGATTGTTACAAGATTCCCGAGATTATATACTAATATAATCTTGGAGCAAGAAG

ATCACGTACTAAAGGTTATTGAAAAACTCGCTAATAAGGCAGATAAACCGGTTAGTATTG

CTAAACCATACCTTGAATTTTCCCTGCCTGAGGGTCATAGAGTTGCAGCTACAGTATCTC

GTGAGGTTTCATTACCTGGTTCCACATTTGATATAAGGAAATTCCCACTAAAGCCAATAA

G

>s15_alpha_v1_1504_Frag 638164210 Alpha-mannosidase

TTAAGGCATTCGCAACTCATAAGGTTTTCTGGAACGACACTAATAAATTCCCATACAACG

TTTTTAAGTGGGTAGGACCAAACGGCGATTACTTACCAGCAATAGCTTTTGGGCATGGAA

AGGGTGGTTATAACTCAGACTTCTCGGCCTCTAGTGTTTTAGAGCAATATAATAATTGGG

CTCAAAAGGATCAACCAATGCTATACTCTTACGGATACGGTGATGGCGGTGGAGGACCAA

ATGAGGATATGTTGATTAGAGCTGAAGCAGTAAACCTTTTACCAATATTACCCAAGGTTG

AACTAAGTGGTGTAAATAGCTATATACAAAGGATAGTTCCAGTAGAAGAGTGGAGAGGAG

AACTATATTTAGAAACTCATAGGGGTGTATTGACCTCTCATTCAAAGATGAAATTGCTTA

ATAGAAGTGCAGAGATTGCGTTAAGAGAAGCCGAGCTATGGAGCACTTTAGCTAGGACTT

ATGATAAAGAAGTTTTTACAAAACTATGGAAAGTTGTATTAAAGGATCAGTTCCATGACG

TCTTACCTGGATCGGCGATAAAAGACGTTTATAAGGTGGCTTATCAAGAGTTAGAAGAAG

TGATAAATAAGGCTAATAATGTTGCGAGTGAAGCGATGCAGAAGTTAGTTGGTGGTAGCG

GAGATAAGACGTTTGTGTTTAATTCACTGTCATGGGATAGGGAGGAATACATTGAGGCTG

ACGGTAAGTTGGTTAAGGTTAGAGTCCCATCAGTGGGATTCTCCTTGTTGGAACCAGTGG

AGGTTAGAGATAAAGCCGTCATTAATGAAAATAACGCTGAATACCTTGTGGAGAATAAAT

ATTTTAGAGTAAGAATAAGTAAAAGTGGGCAAGTTCTCTCACTATTTGATAAAGAGGCAA

ATAGGGAAGTATTAAGGGATAAGAGCAACCTATTAATAGCTTACGAGAATATACCAGGTT

GGGCTGACGCGTGGGATATCGAGAAGGGATTTGAGGATAGAAGTTTCGAAATTAGGGCCT

CCTCATCTGAAATAGTCAATAATGACGGGATTGTGGCTTCAATTAAATTTACTTATAAAT

TTAGGAGATCAGAAATTATTCAAATTGTCCGTGTTTATGCTGATAGTAGAAGGATAGATT

TCATCACTACGCTAAGGATGAGGGATAGAGAACTGTTGGTGAAGAGCTGGTTTAATTTTG

ACTTAAACGTGGAAAGGGCAGTTTCCGATATTCCCTTTGGAGTTGTGGAAAGGTTCACTT

GGAGTAATACCAGTTGGGATAAGGCTAGGTTTGAGGTTCCAATTCAAAAGTTTGTTGATT

TCTCTGAAAGTGAGTATGGTGCCGCATTACTTAATAACGGCAAGTATGGGGCAACGTTAA

GAGGTTCTTCAGTTGGTTTAAGTTTGACTAAAACTCCGATATATCCAGATCCATCTACCG

ACCTTGAAGAGGTCACTTTCATTTACTCATTATATCCTCATATTGGTGATTGGAAGAGGG

CTGA
