# Supplementary material for: Quantitative Transcriptomics Reveals the Growth- and Nutrient-Dependent Response of a Streamlined Marine Methylotroph to Methanol and Naturally Occurring Dissolved Organic Matter
Source: mBio. 2016 Nov 22;7(6):e01279-16. doi: 10.1128/mBio.01279-16 (PMC5120137; doi:10.1128/mBio.01279-16)
Supplement: Figure S1 — Strain NB0046 grown in seawater medium amended with vitamins and inorganic nutrients and in different-size cultivation chambers. Well, 1 ml of culture in a 2-ml well of a 48-well polycarbonate plate. Tube, 8 ml of culture in a 10-ml polycarbonate test tube. Download [file mbo006163064sf1.pdf]

**Figure S1.**

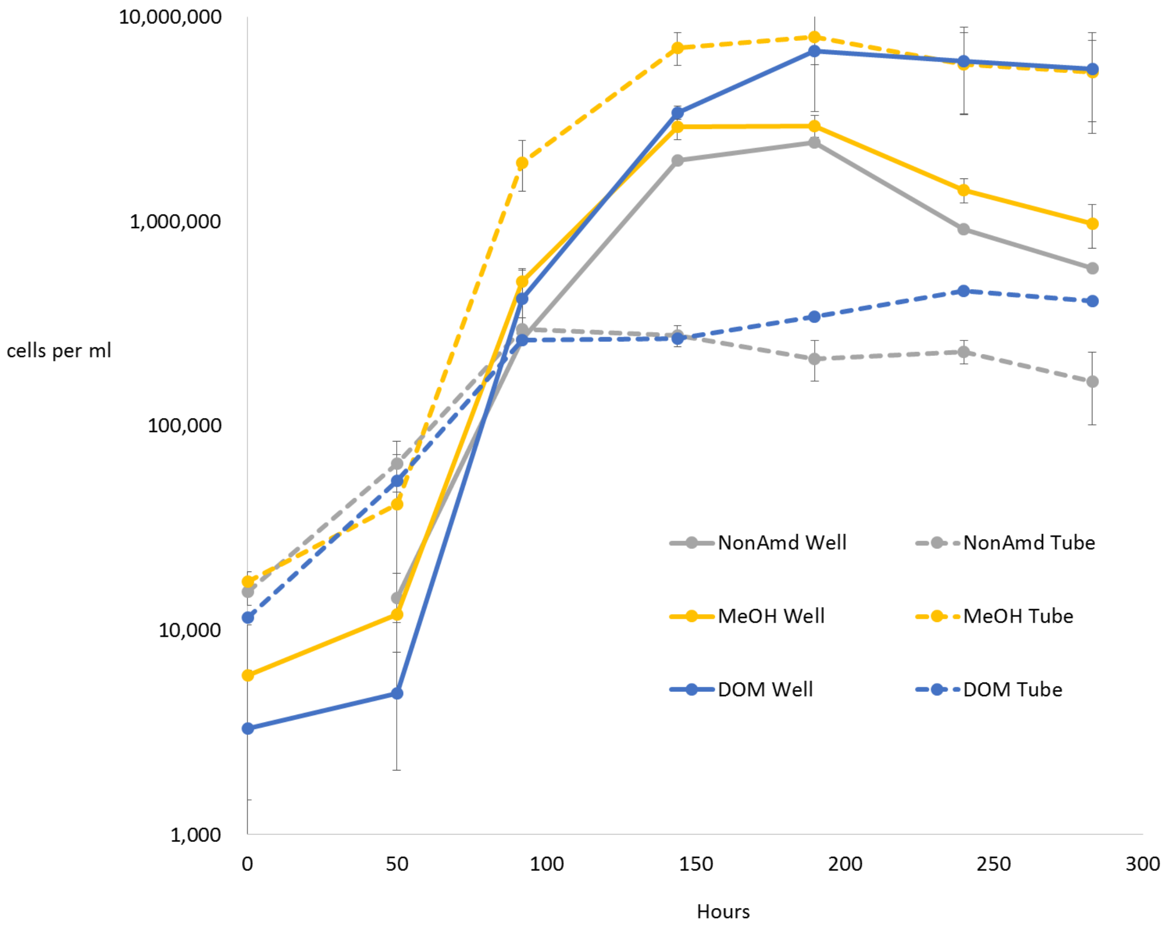


Strain NB0046 growth in seawater medium amended with vitamins and inorganic nutrients and in different sized cultivation chambers. 'Well' = 1ml of culture in a 2ml well in a 48-well polycarbonate plate. 'Tube' = 8 ml of culture in a 10ml polycarbonate test tube.
