# Supplementary figures and images for: Quantitative Transcriptomics Reveals the Growth- and Nutrient-Dependent Response of a Streamlined Marine Methylotroph to Methanol and Naturally Occurring Dissolved Organic Matter
Source: mBio. 2016 Nov 22;7(6):e01279-16. doi: 10.1128/mBio.01279-16 (PMC5120137; doi:10.1128/mBio.01279-16)

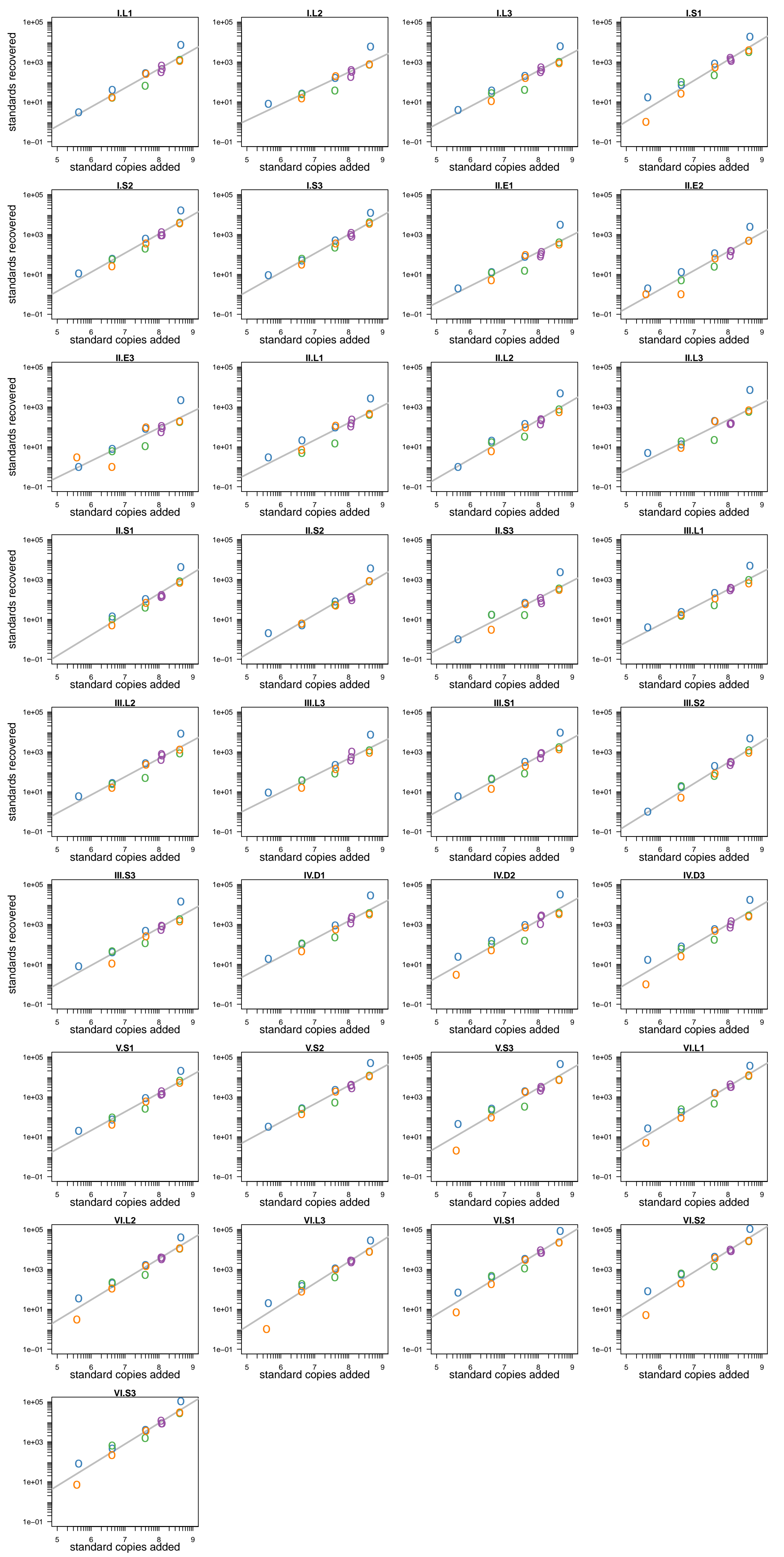

Supplement: Figure S2 — Log-log plot of the recovery of internal standards in the sequence libraries versus the number of standards added for samples collected under all of the regimes used. Colors and symbols are described in Fig. 3. The regime and transcriptome sampled growth phase are indicated above the plot in the format regime number, growth phase, and replicate number. The fitted linear regression line is gray. Download [file mbo006163064sf2.pdf]
